# Archean eclogite-facies oceanic crust indicates modern-style plate tectonics

**DOI:** 10.1073/pnas.2117529119

**Published:** 2022-04-04

**Authors:** Wenbin Ning, Timothy Kusky, Lu Wang, Bo Huang

**Affiliations:** ^a^State Key Laboratory of Geological Processes and Mineral Resources, Center for Global Tectonics, School of Earth Sciences, China University of Geosciences, Wuhan 430074, China;; ^b^Badong National Observation and Research Station of Geohazards, Three Gorges Research Center for Geo-Hazards, China University of Geosciences, Wuhan 430074, China

**Keywords:** eclogite, subduction, Archean, Precambrian tectonics, North China Craton

## Abstract

Establishing when modern-style plate tectonics with deep subduction began on Earth is one of the biggest questions in geosciences today. A lack of Archean age (>2.5 billion y ago [Ga]) eclogites or eclogite-facies crustal rocks (the high-pressure equivalent of basalt or gabbro) has led to an assertion that modern plate tectonics did not operate in the Archean. Here, we report eclogite-facies garnet clinopyroxenite associated with metagabbro in 2.52- to 2.53-billion-y-old ophiolitic mélange in the northern Central Orogenic Belt (COB) within the North China Craton. The garnet clinopyroxenites with normal mid-ocean ridge basalt (N-MORB) geochemical signatures are relicts of oceanic crust, recording peak eclogite-facies metamorphic assemblages indicating conditions of 792 to 890 °C/19.8 to 24.5 kbar, supported by abundant exsolution microstructures in garnet and clinopyroxene. Zircon U-Pb dating of the metagabbros and a granitic dike cross-cutting the metamorphic layering of the metagabbro constrain deformation and eclogite-facies metamorphism to >2.47 Ga. This finding implies that Archean oceanic crust was subducted to at least 65 to 70 km at the end of the Archean. Together with other asymmetric subduction records in the COB, it is inferred that modern-style plate tectonics evidenced by deep and asymmetric subduction along the circa 1,600-km-long orogen was operating at least by the end of the Archean era, when the planet was making a transition to the Proterozoic, witnessing the Great Oxidation Event, widespread emergence of continents, and development of crown node eukaryotic species on a more habitable planet.

## Significance

The onset time of plate tectonics is highly debated in the Earth sciences. A key indicator of modern-style plate tectonics, with deep subduction of oceanic plates, is the presence of eclogite (oceanic crust metamorphosed at high-pressure and low-temperature) in orogenic belts. Since no orogenic eclogites older than 2.1 billion y are currently documented, many scientists argue that modern plate tectonics started only 2.1 billion y ago (Ga). We document an Archean orogenic eclogite, providing robust evidence that subduction of oceanic crust reached to at least 65 to 70 km in depth at circa 2.5 Ga. This extends the known age of subduction-related eclogite-facies metamorphism back 400 My, showing that modern-style plate tectonics operated by the close of the Archean.

Plate tectonics is the major mechanism for heat dissipation from Earth's interior and is the main mode of interaction between the surface and deep Earth, which shapes the continents we live on today (e.g., refs. 1 and 2). The style of plate tectonics likely changed through time, with an early generally “warmer” intraoceanic plate subduction regime giving way to a modern generally “colder” style of intraoceanic and continental margin subduction (2). However, determination of when modern-style plate tectonics was established on Earth has been the focus of heated debate (3–13). Based on diverse constraints from structural geology, geochemistry, petrochronology, metamorphic geology, and numerical modeling, a wide range for the onset time of modern plate tectonics has been suggested, varying from Hadean (1, 3, 4), Eoarchean (2, 5), Mesoarchean (8, 9), or Paleoproterozoic (10) to Neoproterozoic (11) or even Cambrian (12). One of the major arguments against modern-style subduction in the Archean has been the apparent lack of Archean eclogites or eclogite-facies crustal rocks (oceanic crust including basalt or gabbro metamorphosed to high pressures (HPs) at low to moderate temperatures) (e.g., refs. 11–13), which is considered to be unequivocal evidence for the operation of modern-style plate tectonics (7, 10, 11), characterized by large lateral motions of rigid plates with deep and cold asymmetric subduction as documented along many Phanerozoic convergent plate margins (14).

**Fig. 1.**
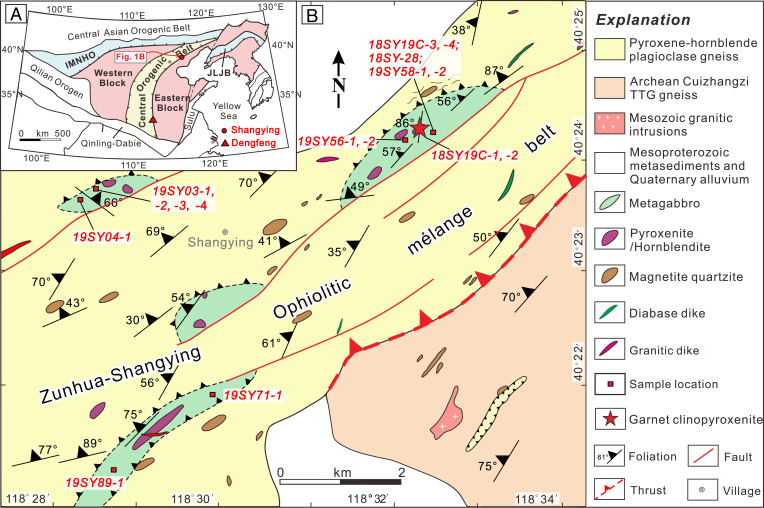
(*A*) A geological map showing the tectonic divisions of the NCC (17). The location of the Dengfeng complex and Shangying rock units along the eastern margin of the COB are labeled. Note that the Dengfeng complex has been documented to preserve a late Archean spatially and temporally linked paired metamorphic belt (21). (*B*) A detailed geological map of Shangying rock units. Note that the garnet clinopyroxenites are enclosed in the metagabbro blocks within the pyroxene–hornblende plagioclase gneiss, which is part of the Archean Zunhua–Shangying ophiolitic mélange belt (*SI Appendix*, Fig. S1*B*). IMNHO, Inner Mongolia–Northern Hebei Orogen; JLJB, Jiao–Liao–Ji Belt.

The North China Craton (NCC) has a prolonged geological history from ∼3.8 billion y ago (Ga) to the present (15–17), providing fragments of a record of early Earth geodynamics. The Precambrian basement of the NCC is divided into four tectonic units, including the Eastern Block, the Western Block, an intervening orogenic belt, and the Inner Mongolia–Northern Hebei Orogen in the north (Fig. 1*A*). The circa 1,600-km-long Central Orogenic Belt (COB) welded the Eastern Block and an intraoceanic arc terrane together, but remains controversial in its collisional age as either end Neoarchean or Late Paleoproterozoic (e.g., refs. 17 and 18). However, there is growing evidence recorded in the Dengfeng, Zanghuang, Eastern Hebei, and Jianping complexes within the COB that indicates that the orogen formed by an end Neoarchean arc–continent collision (*SI Appendix*, Fig. S1*A*). Preserved structural and lithic records of this collision include a series of Archean ophiolitic (oceanic crust and mantle) fragments and tectonic mixtures of rocks known as mélanges (19), exotic belts of forearc magmatic rocks that preserve Archean subduction initiation sequences (20), paired metamorphic belts (21), ultrahigh-pressure (UHP) metamorphic crustal minerals (22), forearc and accretionary complexes (23), and Alpine-style subhorizontal arc–affinity nappes emplaced over a continental margin (24), all of which are diagnostic indicators of asymmetric Archean subduction and large plate translations and analogous to modern subduction at convergent tectonic margins. Nevertheless, the deep subduction of Archean oceanic crust demarked by hallmark vestiges of orogenic eclogites has not been documented in the NCC or anywhere worldwide.

Here, we report a discovery of eclogite-facies garnet clinopyroxenite in the Archean Zunhua–Shangying ophiolitic mélange of the COB, NCC. Through comprehensive metamorphic petrological, geochemical, and geochronological analyses, we show that the garnet clinopyroxenite with oceanic gabbro affinity experienced eclogite-facies metamorphism at the end of the Neoarchean, and peak metamorphic pressure-temperature (P-T) conditions of 792 to 890 °C/19.8 to 24.5 kbar are retrieved, indicating that Archean oceanic crust was subducted to at least 65 to 70 km. This result provides direct petrological evidence of deep, relatively cold subduction of oceanic crust during the Archean era. Integrated with other asymmetric subduction records preserved in the complexes within the COB, we suggest that the circa 1,600-km-long subduction zone is evidence of large-scale horizontal plate motion and deep subduction at the end of Archean, representing the operation of modern-style plate tectonics.

## Archean Zunhua–Shangying Ophiolitic Mélange

The Zunhua–Shangying ophiolitic mélange is a Neoarchean ophiolitic mélange (20, 25) in which oceanic affinity rock assemblages were emplaced over the Eastern Block of the NCC at the end of the Archean and the dawn of the Proterozoic (*SI Appendix*, Fig. S1). The ophiolitic mélange contains a large number of metamorphosed mafic–ultramafic blocks of oceanic crust and mantle affinity (gabbro, diabase, basalt, peridotite, pyroxenite, podiform chromitite, chert/banded iron formation) sheared together in a matrix of strongly deformed metasedimentary rocks, preserving typical “block-in-matrix” mélange relationships (19, 26). The Shangying rock units mainly consist of metamorphosed pyroxene–hornblende plagioclase gneiss, gabbro, garnet clinopyroxenite, cumulate hornblendite and pyroxenite, and pyroxene magnetite quartzite (Fig. 1*B*). The age of the mélange belt is well constrained, with blocks yielding ages obtained from multiple isotopic systems (U-Pb, Pb-Pb, Lu-Hf, Re-Os) ranging from 2.69 to 2.52 Ga, most being members of the ophiolite suite with ages ranging between 2.55 and 2.52 Ga (20, 25). Detrital zircons in sedimentary lenses and the matrix of the mélange have U-Pb ages between 2.87 and 2.52 Ga, with the youngest cluster of ages being 2,543 ± 15 Ma (20), and metamorphic rims on zircons indicate that postdeformation thermal effects lasted until 2,467 ± 27 Ma. Tectonic events associated with mélange formation ended before this time, as confirmed by cross-cutting granitic dikes with ages of 2,458 ± 17 Ma (25). Below, we report geochronological data from the block in mélange containing the garnet clinopyroxenite described in this contribution.

**Fig. 2.**
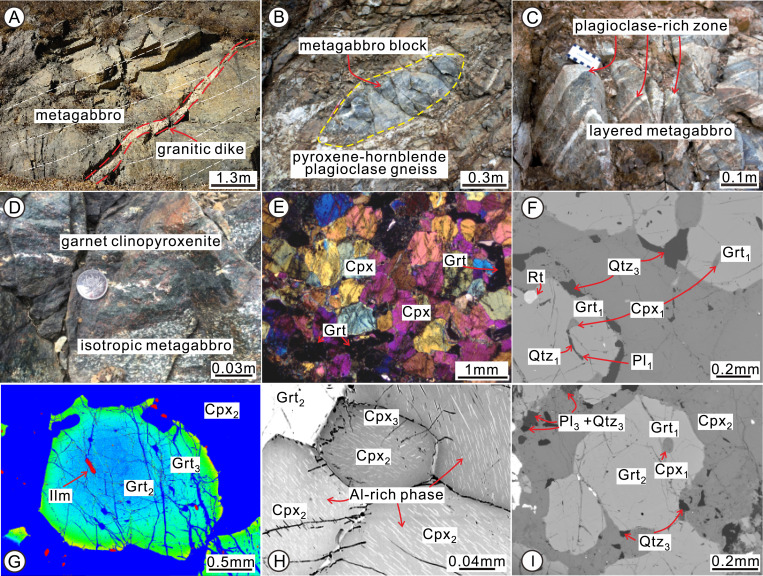
(*A*) A granitic dike (outlined by red dashed lines) cross-cuts the internal layering (white dashed lines) of the metagabbro. (*B*) Metagabbro blocks within the pyroxene–hornblende plagioclase (Pl) gneiss. (*C* and *D*) Layered and isotropic metagabbro, which includes garnet (Grt) clinopyroxenite. (*E*) Clinopyroxene (Cpx) grains in garnet clinopyroxenite have typical triple-junction textures with dihedral angles of ∼120°, indicating that these grains were in thermodynamic equilibrium during recrystallization. (*F*) A BSE image showing different stages of quartz (Qtz), Grt, Cpx, and Pl. (*G* and *H*) High-contrast BSE images of Grt and Cpx (*G* is in color scale) displaying zoning texture with the exsolved Al-rich phase on the Cpx surface. Note that the ilmenite (Ilm) within the Grt is likely altered from rutile (Rt) by the metasomatism with retrograde fluid through the cracks within Grt. (*I*) BSE image showing different stages of Grt and Cpx with small Qtz and Pl around the euhedral garnet.

The metagabbros in Shangying have isotropic and layered field characteristics. The layered metagabbro contains layers of plagioclase-rich and garnet-rich assemblages, while the isotropic metagabbro is generally massive but locally preserves a faint layering (Fig. 2 and *SI Appendix*, Fig. S2). Massive-structured garnet clinopyroxenite is enclosed in the weakly foliated isotropic metagabbro with distinct petrological contacts. It includes garnet (35 to 40 vol %), clinopyroxene (50 to 55 vol %), ilmenite (∼5 vol %), and magnetite (2 to 3 vol %), with accessory plagioclase, quartz, rutile, and titanite. More detailed field and micropetrographic features of garnet clinopyroxenite are depicted in Fig. 2 and *SI Appendix*, Figs. S2 and S3.

The garnet clinopyroxenite preserves mineral assemblages formed during four metamorphic stages (M1 to M4). M1 is demarcated by rounded, fine-grained inclusions of clinopyroxene (Cpx_1_), plagioclase (Pl_1_), and quartz (Qtz_1_) encapsulated in the cores of some euhedral garnets (Fig. 2*F* and *SI Appendix*, Fig. S3*H*), indicating that the inclusion-rich garnet core domains (Grt_1_) in direct contact with the inclusions are in chemical equilibrium with the enclosed mineral phases (i.e., Cpx_1_ + Pl_1_ + Qtz_1_). Therefore, this mineral assemblage is interpreted to have been produced by prograde metamorphism (M1). M2 is marked by large matrix clinopyroxene and garnet crystals that show compositional zoning under element mapping and backscattered electron (BSE) imagery (Figs. 2 *G* and *H* and *SI Appendix*, Fig. S4). Thus, clinopyroxene compositional cores (Cpx_2_) and other relatively clean garnet compositional cores (Grt_2_; in contrast to the inclusion-rich Grt_1_ cores) are considered to record the peak metamorphism (M2) (Fig. 2 *G* and *H*). The compositions of Grt_1_ and Grt_2_ present no prominent differences, but their shapes and sizes and the nature of their mineral inclusions are different. In addition, relatively idioblastic, clean garnet phenocrysts (Grt_2_ in the core) typically have compositional rims (Grt_3_). The compositional rims (Grt_3_, Cpx_3_) plus the small plagioclase (Pl_3_) and quartz grains (Qtz_3_) surrounding the garnet record the first step of the retrograde metamorphism (M3) (Fig. 2 *F*–*I*). The final retrograde assemblage (M4) at greenschist–amphibolite facies is recorded by actinolite (Act_4_), chlorite (Chl_4_), epidote (Ep_4_), and magnetite (Mag_4_). Many large ilmenite crystals either developed along the garnet grain boundaries or are preserved as inclusions within garnet (*SI Appendix*, Fig. S3 *B*, *C*, and *J*). They also appear as four types of bi- and multiphase inclusions within the garnet and clinopyroxene, including titanite + magnetite + ilmenite, magnetite + ilmenite, rutile + titanite + ilmenite, and rutile + ilmenite (*SI Appendix*, Fig. S3 *D*–*G*).

## P-T Conditions and Eclogite-Facies Metamorphism

**Fig. 3.**
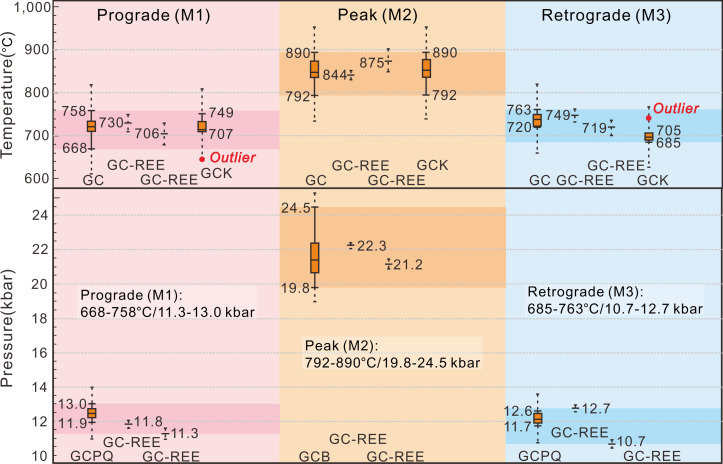
Plots showing the P-T conditions of garnet clinopyroxenite at different metamorphic stages. The P-T conditions were estimated by various geothermobarometers labeled in the plot. The boxes represent the interquartile range of the dataset of retrieved temperature or pressure. The outliers marked by red dots are far away from other retrieved temperature or pressure values and excluded in the final evaluation of the P-T conditions. The maximum and minimum values of the credible P or T conditions are labeled at the outward extension of the boxes (solid line segment). Dashed lines extending from each P-T box represent the SEs of the employed geothermometers or geobarometers. The geothermobarometers include garnet–clinopyroxene Fe-Mg exchange geothermometer (GC) (56), rare earth element (REE)-based garnet–clinopyroxene thermobarometer (GC-REE) (27), garnet–clinopyroxene Fe-Mg geothermometer (GCK) (57), garnet–clinopyroxene–plagioclase–quartz geobarometer (GCPQ) (58), and garnet–clinopyroxene geobarometer (GCB) (59).

Representative major and trace elements of the garnet and clinopyroxene and major elements of plagioclase from the first three metamorphic stages within garnet clinopyroxenite were analyzed. The metamorphic P-T conditions of the different metamorphic phases described above were determined by various geothermobarometers. The results employing the REE-based garnet clinopyroxene thermobarometer (27) are shown in *SI Appendix*, Fig. S5 and Table S5, and results of all the geothermobarometry employed in this study and the calculated P-T conditions are compiled in Fig. 3 and *SI Appendix*, Tables S4 and S5.

Fig. 3 shows the temperature and pressure conditions estimated by applying all applicable thermobarometers on the 6 garnet–clinopyroxene–plagioclase–(quartz) mineral pairs of the M1 phase, 11 garnet–clinopyroxene mineral pairs of the M2 phase, and 6 garnet–clinopyroxene–plagioclase–(quartz) mineral pairs of the M3 phase (*SI Appendix*, *Supplementary Text* has a detailed description of mineral chemistry and the calculation of P-T conditions). M4 lacks sufficiently diverse mineral species for calculation of P-T conditions, but the assemblage is consistent with a retrograde amphibolite–greenschist path. Considering all the results of the thermobarometric calculations in Fig. 3, the most reliable range of temperature and pressure for each metamorphic stage is calculated to be 668 to 758 °C/11.3 to 13.0 kbar for M1, 792 to 890 °C/19.8 to 24.5 kbar for M2, and 685 to 763 °C/10.7 to 12.7 kbar for M3.

The peak metamorphic P-T conditions of 792 to 890 °C/19.8 to 24.5 kbar show that the protolith of the garnet clinopyroxenite experienced eclogite-facies metamorphism, with the P-T conditions indicating pressures equivalent to subduction to at least ∼65 to 70 km. However, the jadeite molecule of the clinopyroxene in garnet clinopyroxenite is absent, which might be attributed to the low Na_2_O content of its protolith, preventing the growth of Na-rich omphacite. However, this does not affect the P-T calculations. Details of the effect of low Na (jadeite) are discussed in *SI Appendix*, *Supplementary Text*.

The eclogite-facies metamorphism is also indicated by several exsolution textures of representative minerals (*SI Appendix*, Fig. S3 *K*–*M*). Rutile lamellae exsolved from garnet are present in the garnet clinopyroxenite (*SI Appendix*, Fig. S3*K*). These aligned rutile needles range in diameter from a few hundred nanometers to a few micrometers and are hundreds of micrometers in length, implying decreased pressure and temperature due to exhumation from HP–UHP metamorphic conditions (28). In addition to the exsolution lamellae in garnet, the clinopyroxene crystals in the garnet clinopyroxenite preserve oriented exsolved needles of garnet (*SI Appendix*, Fig. S3 *L* and *M*). Textures of garnet exsolved from clinopyroxene have been widely reported in eclogites and pyroxenites in many UHP–HP metamorphic complexes (e.g., ref. 29). The exsolution of garnet from clinopyroxene in the garnet clinopyroxenite xenoliths from the Xuzhou region (near the Sulu UHP metamorphic terrane, central eastern China) is considered to be caused by reactions driven by decreasing temperature from ∼950 to 1,100 °C down to 620 to 780 °C at a nearly constant pressure from ∼15 to 20 kbar to 18 kbar (29). The composition of the exsolved garnet rods is similar to that of the garnet rims (M3 stage) from this study; thus, garnet lamellae probably formed during the cooling of the rock after the peak eclogite-facies metamorphism. M4, recorded by actinolite (Act_4_), chlorite (Chl_4_), epidote (Ep_4_), and magnetite (Mag_4_), represents further cooling during exhumation through amphibolite and greenschist facies.

## Eclogite-Facies Metamorphism at the End of the Archean

Zircons from two metagabbros (containing the eclogite-facies garnet clinopyroxenite) and one granitic dike cutting through the metagabbro (Fig. 2*A*) were separated for U-Pb geochronological study (*SI Appendix*, Fig. S6). The magmatic zircons within two metagabbro samples yield weighted mean ^207^Pb/^206^Pb ages of 2,528 ± 30 and 2,522 ± 15 Ma (ages are reported with 2σ), interpreted as the crystallization age of their protolith (*SI Appendix*, *Supplementary Text* has a detailed description of zircon U-Pb geochronology and Hf isotopic composition). This age is consistent with those from similar rocks from other areas in the Zunhua–Shanying mélange belt (20, 25, 30). Therefore, the crystallization age of the protolith of the metagabbros is constrained between 2.52 and 2.53 Ga.

Both metagabbros record an early Paleoproterozoic metamorphic age (2,471 ± 38 and 2,474 ± 26 Ma), consistent with widely reported metamorphic ages in the COB (15, 30). One of the metagabbros also yields 2 zircon rims (of 13 zircon rims) with a late Paleoproterozoic metamorphic age of 1,823 ± 56 Ma, in accord with the circa 1.81-Ga metamorphic age locally reported in the Eastern Hebei complex (20, 30) from a younger tectonothermal event under a different tectonic setting.

**Fig. 4.**
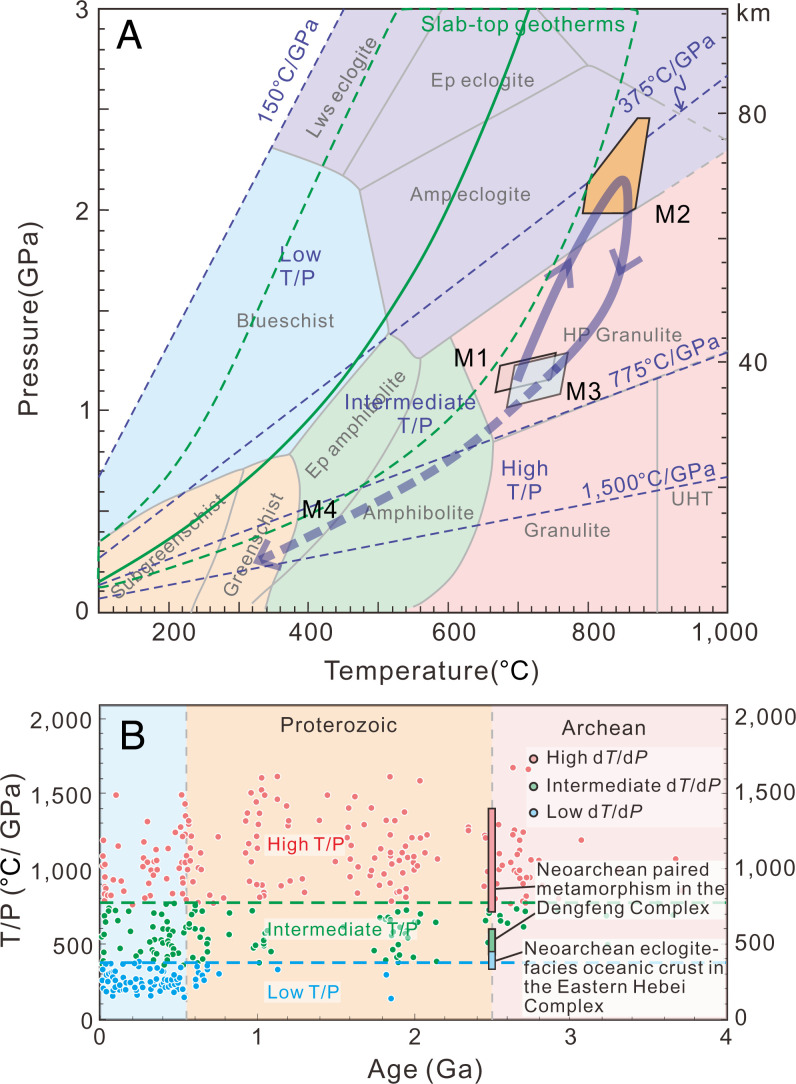
(*A*) Retrieved P-T-t path and (*B*) geothermal gradient (degrees Celsius per gigapascal) of the Shangying garnet clinopyroxenite. The metamorphic facies (60) and the slab-top geotherms of Neoproterozoic to Phanerozoic subduction zones (bounded by the light green dashed lines) (37) are shown. The high, intermediate, and low dT/dP areas delineated by 1,500, 775, 375, and 150 °C/GPa are defined based on the global metamorphic P-T dataset (11, 45). dT/dP, differential temperature/differential pressure; UHT, ultra-high temperature. Both the Dengfeng complex and the Eastern Hebei complex are located along the eastern margin of the COB (*SI Appendix*, Fig. S1*A*), NCC. The Late Archean spatially and temporally linked paired metamorphism documented from the Dengfeng complex (21) and the contemporaneous eclogite-facies oceanic crust in the Eastern Hebei complex (this study), indicate that the modern plate tectonics, characterized by asymmetric and deep subduction, operated at the end of Archean.

The postkinematic granitic dike cross-cutting the internal layering of the metagabbro containing the eclogite-facies garnet clinopyroxenite yields a crystallization age of 2,468 ± 23 Ma, further constraining the deformation, eclogitic metamorphism, and minimum timing of exhumation of the garnet clinopyroxenites to be greater than 2.47 Ga. Thermodynamic forward modeling for high-grade rocks (31), including high- and ultrahigh-P eclogite (32), suggests that zircons grow mainly during late-stage exhumation and cooling, thus postdating the metamorphic peak (Fig. 4). Therefore, we consider that the eclogite-facies metamorphism resulting in the formation of garnet clinopyroxenite occurred between 2.53 and 2.47 Ga at the end of the Archean eon.

## Relict of Oceanic Crust Origin for the Metagabbro and Garnet Clinopyroxenite

The metagabbros are characterized by slightly negative to positive Nb anomalies (Nb/Nb* = 0.78 to 1.34, 1.14 on average), suggesting that crustal contamination did not play a significant role in the processes of magma crystallization and emplacement (33). They have slightly negative to positive Zr, Hf, and Ti anomalies, and most samples possess deficient light rare earth elements (LREEs), showing similar geochemical affinities with typical normal mid-ocean ridge basalt (N**-**MORB) (*SI Appendix*, Fig. S7). Some metagabbros display relatively enriched LREE ([La/Yb]_cn_ = 1.09 to 2.77) and significantly positive Pb anomalies (Pb/Pb* = 1.10 to 7.08), indicating that their protoliths were metasomatized by subduction-related fluids/melts, perhaps in the subduction channel. They have magmatic zircons with ε_Hf_ values ranging from + 3.6 to + 7.2, and their Hf model ages (T_DM1_) are similar to the formation age, consistent with their derivation from the depleted mantle. Moreover, on the Nb/Yb-Th/Yb and Zr-Nb-Y diagrams (*SI Appendix*, Fig. S8), most of the metagabbros display N-MORB geochemical affinity. In addition, the Shangying metagabbros are layered in some outcrops, including layers of plagioclase-rich assemblages, consistent with the characteristics of oceanic gabbro (34). Therefore, we suggest that the metagabbros represent remnants of mid-to-lower units of oceanic crust derived from partial melting of depleted mantle.

The garnet clinopyroxenites have relatively low contents of MgO (8.36 to 9.12 wt %), Cr (229 to 253 ppm), and Ni (102 to 172 ppm) and relatively high Al_2_O_3_ (11.39 to 14.46 wt %) contents, precluding an origin as a continental (intraplate) layered intrusion, further illustrated by the trace element (Nb, Zr, Y) discriminant plot (*SI Appendix*, Fig. S8*B*). In addition, pyroxenites with a metasomatic origin typically occur in mantle peridotite in the form of veins rather than massive bodies (35), which is different from the occurrence of the Shangying garnet pyroxenite. The Shangying garnet clinopyroxenite has a granoblastic metamorphic texture with typical grain boundary dihedral angles of ∼120°, and some mineral aggregates show a fine-grained, xenomorphic texture, indicating that the present minerals may be formed by the breakdown of former minerals, caused by later metamorphism during static annealing. In addition, the metagabbros and garnet clinopyroxenites have compositional trends in terms of major elements and immobile trace elements (*SI Appendix*, Fig. S9), indicating they may possess a close genetic relationship. On the major elements versus MgO diagrams (*SI Appendix*, Fig. S10), compositions of the garnet clinopyroxenites resemble metamorphosed oceanic gabbros rather than continental cumulate mantle pyroxenites. We conclude that the Shangying garnet clinopyroxenite is a relict of oceanic crust that crystallized at an oceanic spreading center at 2.52 to 2.53 Ga, was metamorphosed to eclogite facies deep in a subduction zone between 2.53 and 2.47 Ga, and then was exhumed to upper crustal levels where it was intruded by granite dikes at 2.46 Ga. This marks the end of the Archean and the dawn of the Proterozoic, representing a harbinger to the start of the Great Oxidation Event that changed Earth’s surface and biological systems, making the planet habitable.

## Modern-Style Plate Tectonics Operated by the Late Archean

The garnet clinopyroxenites contain neither orthopyroxene nor amphibole nor any other hydrous minerals, showing that they formed in dry eclogite facies, inherited from a dry protolith, rather than epidote eclogite-facies or amphibole eclogite-facies conditions (Fig. 4*A*). The retrieved clockwise hairpin-type P-T path is tight but similar to some of those recorded by many garnet clinopyroxenites and eclogites from Paleoproterozoic to Phanerozoic subduction–collision settings (14, 36). The peak metamorphic conditions of the garnet clinopyroxenite correspond to geothermal gradients of 10 to 13 °C/km (323 to 449 °C/GPa), slightly higher than slab-top geotherms of most Neoproterozoic–Phanerozoic subduction zones (mostly below 10 °C/km) (37), and reflect slightly warmer geotherms in this particular latest Archean subduction zone.

Previous work suggested that the late Neoarchean–early Paleoproterozoic metamorphism was related to subduction and subsequent arc–continent collision between the Wutai/Fuping arc terrane and the Eastern Block of the NCC, resulting in the formation and emplacement of the Zunhua–Shangying ophiolitic mélange (19, 20, 25). The garnet clinopyroxenite and metagabbro with N-MORB compositions occur as blocks and elongated tectonic slices in the Zunhua–Shangying ophiolitic mélange, which may represent relicts of subducted Archean oceanic crust tectonically emplaced over the western margin of the Eastern Block of the NCC.

Eclogite in Phanerozoic orogens is widely regarded to represent exhumed fragments of subducted continental or oceanic crust or alternatively, could theoretically be formed at deeper levels of thick basaltic crust generated by nonplate-tectonic processes (38). However, our detailed field mapping results from the Zunhua–Shangying ophiolitic mélange (20, 25) show the chaotic juxtaposition of metagabbro, garnet clinopyroxenite, metabasalt, metaperidotite, and metapelite (Fig. 1), with different sources and metamorphic conditions, revealing the structural and metamorphic diversity and complexity, which are analogous to subduction channels (*SI Appendix*, Fig. S11) in younger orogens (14, 26). In addition, the chemical composition of the protoliths is N-MORB, not a continental intrusion. Therefore, the Shangying garnet clinopyroxenite with eclogite-facies peak metamorphism indicates that Archean oceanic crust was subducted to at least 65 to 70 km, and then exhumed through a subduction channel, where it was structurally fragmented into isolated blocks forming components of the ophiolitic mélange (*SI Appendix*, Fig. S11). Mélanges of the COB are varied in their components along strike (19), from the dominantly mafic/ophiolitic variety at Shangying to the ophiolitic UHP podiform chromite type at Zunhua to dominantly metasedimentary mélanges at Zanhuang and Dengfeng, where a classical orogenic paired metamorphic belt relationship is preserved (21) (*SI Appendix*, Fig. S1*A*). All of the exhumed mélanges were intruded at 2.47 Ga by granitic dikes, providing an upper limit for the timing of deformation and peak metamorphism along the entire plate-scale 1,600-km-long orogenic belt.

This discovery documents the oldest eclogite-facies garnet clinopyroxenite within tectonic mélange preserved in an arc–continent collisional orogen on Earth, extending the known age of subduction-related HP metamorphism back by more than 400 My. The next oldest confirmed orogenic eclogites or orogenic eclogite-facies rocks are much younger and found in Paleoproterozoic orogens, including the 1.8-Ga Trans-Hudson orogen of North America (39), the 1.9- to 2.0-Ga Ubendian–Usagaran Belt of Tanzania (40), the 2.1-Ga metamorphism in the Kasai Block of the Congo Craton (41), and the 2.09-Ga Eburnian–Transamazonian orogen of Cameroon (42). A reported medium-temperature Archean eclogite from the Belomoran massif of the Fennoscandian Shield (43) was later shown to have reached eclogite facies only at circa 1.9 Ga (44). The rarity of Archean eclogite perhaps results from a preservation/exhumation bias (45). The higher Archean geothermal gradient may have led to more frequent slab break off (46), and later overprinting metamorphism or deformation and fluid/melt metasomatism may also make the early eclogite-facies rocks difficult to preserve. However, in this study, the dry eclogite-facies garnet clinopyroxenites are contained in metagabbro blocks of a fluid-rich Neoarchean ophiolitic mélange, which prevented fluid/melt from metasomatizing the clinopyroxenite, resulting in the preservation of the eclogite-facies assemblage. This rheological difference between the dry strong eclogite block, in a weak wet matrix, likely also aided its exhumation in the subduction channel (*SI Appendix*, Fig. S11*C*). This study shows that the preservation of eclogite-facies rocks is critically dependent on having inherited dry rocks, which favors the preservation of the higher-grade metamorphic assemblages (47), and further shows that eclogite-facies rocks were produced on the early Earth and preserved under these special dry conditions. Further work is required on even older samples globally less affected by later overprinting metamorphism or fluid/melt metasomatism to better understand the geodynamics and tectonics of the early Earth.

## Materials and Methods

### Whole-Rock Major and Trace Element Analyses.

Whole-rock powders were made from two garnet clinopyroxenites and 12 surrounding metagabbros within the Zunhua–Shangying ophiolitic mélange using an automated agate or alumina ball mill or by hand using an agate mortar and pestle. The major and trace element analyses of these whole-rock powders were conducted on an X-ray fluorescence spectrometer (Primus II) and with inductively coupled plasma mass spectrometry (ICP-MS; Agilent 7700e), respectively, at the Wuhan Sample Solution Analytical Technology Co., Ltd., and the results are presented in *SI Appendix*, Table S1. The detailed sample digestion procedure for ICP-MS analyses followed the protocols at the State Key Laboratory of Geological Processes and Mineral Resources (GPMR), China University of Geosciences (CUG) (48). The analytical uncertainties on the major and trace element concentrations are generally greater than 1 and 5%, respectively.

### Zircon U-Pb Isotope and Trace Element Analyses.

Zircon grains from metagabbros (18SY19C-3 and 19SY03-1) and granitic dike (18SY-28) were separated following conventional crushing, sieving, magnetic separation, and heavy liquid procedures. Next, these zircons from the same sample were mounted on an epoxy resin target with a 16-mm diameter, which was subsequently polished down to an approximate cross-section so as to clarify their internal structure of zircons under cathodoluminescence (CL) images. Zircon CL images were obtained using an FEI Quanta 450 field emission gun scanning electron microscope connected to a Gatan Mono CL 4+ CL system at the State Key Laboratory of GPMR, CUG. The imaging conditions include an acceleration voltage of 10 kV and a working distance of 13.9 to 14.1 mm. U-Pb dating and trace element analysis of zircon were simultaneously performed on an Agilent 7500a ICP-MS instrument with an ArF excimer laser at the Wuhan Sample Solution Analytical Technology Co., Ltd. The spot size and frequency of the laser were set to 32 µm and 3 Hz, respectively. The detailed configuration conditions and operation procedure followed previous protocols (48). Concordia diagrams and weighted mean age calculations were made using Isoplot _ver4.15 (49).

### Zircon Lu-Hf Isotope Analyses.

Zircon Hf isotopic analyses were carried out on a Neptune Plus Multiple-Collector ICP-MS (Thermo Fisher Scientific) connected with a Geolas HD Excimer ArF laser ablation system (Coherent), which was also hosted at the Wuhan Sample Solution Analytical Technology Co., Ltd. The operating conditions and steps followed the previous protocols reported in this laboratory (50). The laser spot size for this measurement was 44 µm. The off-line selection, integration of background and analytical signals, time-drift correction, and quantitative calibration for trace element analyses were performed with the software ICPMSDataCal (51). The initial ^176^Hf/^177^Hf values of the zircons were calculated using their corresponding ^207^Pb/^206^Pb ages. The present-day ^176^Hf/^177^Hf and ^176^Lu/^177^Hf ratios of chondrite and depleted mantle employed in this study are 0.28772/0.0332 and 0.28325/0.0384, respectively (52, 53). A decay constant (54) for ^176^Lu is 1.867 × 10^−11^ a^−1^. The detailed zircon U-Pb isotopic compositions, trace elements, and Lu-Hf isotopic compositions are presented in *SI Appendix*, Tables S2 and S3, respectively.

### Mineral Major and Trace Element Analyses.

BSE images observations, major elements analyses, and X-ray compositional mapping were conducted on a JEOL JXA-8230 electron probe microanalyzer equipped with four wavelength-dispersive spectrometers at the Center for Global Tectonics, School of Earth Sciences, CUG. A 15-kV accelerating voltage, a 20-nA probe current, and a 1- to 3-μm beam diameter were used during measurement. A series of standards from SPI Co., Ltd. was utilized, and the raw X-ray intensities were corrected by a ZAF (atomic number [Z], absorption, fluorescence) correction procedure (55). All major oxides concentrations accurately reproduced standard compositions within ∼2%, and most major oxides are within ∼1% compared with standard values.

Trace element analyses of the garnet and clinopyroxene in garnet clinopyroxenite were conducted by laser ablation ICP-MS at the Wuhan Sample Solution Analytical Technology Co., Ltd. The operating conditions for the laser ablation system and the ICP-MS instrument and data reduction followed the protocols in the laboratory (50). The ablation spot size was 44 μm, with a laser frequency of 5 Hz. All the acquired data processing and calibration were completed on the software ICPMSDataCal (51). Representative major elements of the garnet, clinopyroxene, and plagioclase from the three metamorphic stages within garnet clinopyroxenite are given in *SI Appendix*, Table S4, and representative trace elements of the garnet and clinopyroxene are given in *SI Appendix*, Table S5.

## Supplementary Material

Supplementary File

## Data Availability

All the data that support the findings of this study are included in the article and/or *SI Appendix*.
