# Supplementary material for: Archean eclogite-facies oceanic crust indicates modern-style plate tectonics
Source: Proc Natl Acad Sci U S A. 2022 Apr 4;119(15):e2117529119. doi: 10.1073/pnas.2117529119 (PMC9169650; doi:10.1073/pnas.2117529119)
Supplement: Supplementary File [file pnas.2117529119.sapp.pdf]

PNAS
www.pnas.org

Supplementary Information for

Archean eclogite-facies oceanic crust indicates modern-style plate tectonics

Wenbin Ning, Timothy Kusky, Lu Wang, Bo Huang

Corresponding authors: Timothy Kusky, Lu Wang

Email: tkusky@gmail.com and wanglu@cug.edu.cn.

This PDF file includes:

Supplementary text: 1. Mineral chemistry;

2. Geothermobarometers and P-T conditions;

3. Zircon U-Pb geochronology and Hf isotopic composition.

Figures S1 to S11

Tables S1 to S5

SI References

Mineral chemistry

Representative major elements of the garnet (M1-M3), clinopyroxene (M1-M3) and plagioclase (M1, M3) from the three metamorphic stages within garnet clinopyroxenite are given in *SI Appendix,* Table S4, and representative trace elements of the garnet, clinopyroxene are given in *SI Appendix,* Table S5. Garnet in sampled garnet clinopyroxenite is dominated by almandine ($X_{Alm} = Fe^{2+}/(Fe^{2+} + Mg + Ca + Mn) = 0.51–0.56$), pyrope ($X_{Prp} = Mg/(Fe^{2+} + Mg + Ca + Mn) = 0.22–0.29$), grossular ($X_{Grs} = Ca/(Fe^{2+} + Mg + Ca + Mn) = 0.18–0.22$) composition with minor spessartine ($X_{Sps} = Mn/(Fe^{2+} + Mg + Ca + Mn) = 0.01–0.02$). High-contrast back-scattered electron (BSE) images (Fig. 2*G*) and compositional profiles (*SI Appendix,* Figs. S4*A*–*4B*) of garnets reveal a small chemical zonation from core (Grt_2) to rim (Grt_3). X_{Prp}, X_{Mg} (= Mg/(Mg + Fe)) monotonically decreases from core to rim, which is compensated by increasing X_{Alm}, and X_{Grs} show nearly no compositional zoning or slightly increased from the core. These compositional trends of garnet run counter to the variation of typical growth zonation (e.g., 1–2), which is inferred as the effect of strong diffusional resorption induced by retrogression. In addition, the outermost narrow rim shows slightly decreased X_{Grs} compared to the rim, indicating a later diffusive re-exchange event. Since the compositions of garnet rims are modified by retrogression, the garnet cores with maximum X_{Mg} value are utilized to calculate the peak P-T conditions.

Diopside is the clinopyroxene in the garnet clinopyroxenite based on the classification of the Morimoto (3) with negligible jadeite fraction (*SI Appendix,* Fig. S4*C*). As demonstrated by high-contrast BSE images (Fig. 2*I*) and compositional profile (*SI Appendix,* Fig. S4*D*), from core (Cpx_2) to rim (Cpx_3), the X_{En} (= $Mg/(Fe^{2+} + Mg + Ca) = 0.36–0.40$) and X_{Mg} (= Mg/(Mg + Fe) = 0.67–0.76) monotonically increase, compensated by decreasing X_{Fs} ($=Fe^{2+}/(Fe^{2+} + Mg + Ca) = 0.12–0.17$) and X_{Wo} ($=Ca/(Fe^{2+} + Mg + Ca) = 0.45–0.48$).

The REE patterns of garnet and clinopyroxene grains (M1-M3) that were used for the P-T estimation in the garnet pyroxenite are shown in *SI Appendix,* Figs. S4*E*–*4F*, and Table S5. Criteria of classification for their metamorphic stages are described in the main text. Garnets exhibit highly enriched HREE patterns with $(Gd/Yb)_N = 0.60–1.00$, negative Eu (Eu/Eu* = 0.64–0.99) and Ce anomalies (Ce/Ce* = 0.14–0.89), and total REE of 24.329–34.655 ppm. There is a small but perhaps insignificant variation in REE concentrations from garnet cores to rims, amounting to less than 10 ppm. The REE patterns of clinopyroxene are opposite to that of garnet, which show enriched LREE and MREE compared to HREE ($(La/Yb)_N = 11.78–39.50$, $(Gd/Yb)_N = 24.09–97.28$, $(La/Sm)_N = 0.21–0.33$) with negative Eu (Eu/Eu* = 0.80–1.03) and positive Ce (Ce/Ce* = 1.08–1.20) anomalies. The total REE contents range from 22.723 to 29.237 ppm.

Feldspars in the garnet clinopyroxenite were also probed for calculating the P-T conditions of prograde

metamorphism (M1) and the retrograde metamorphism (M3). The compositions of plagioclase inclusions in the garnet core (Pl_1) are similar to those of plagioclase around garnet rim (Pl_3). Plagioclases in the prograde metamorphic stage have X_{An} (= Ca/(Ca + K + Na)) values of 0.33–0.37, and X_{Ab} (= Na/(Ca + K + Na)) values of 0.63–0.67. The composition of plagioclase in the retrograde metamorphic stage (M3) varies more widely, with X_{An} values of 0.36–0.41 and X_{Ab} values of 0.59–0.64. However, the X_{Or} (= K/(Ca + K + Na)) values in the two stages of plagioclase are almost zero.

Geothermobarometers and P-T conditions (M1-M3)

Metamorphic temperature and pressure conditions of the garnet clinopyroxenite were determined using various published geothermometers coupled with geobarometers, which together with the calculated P-T conditions are compiled in Fig. 3 and *SI Appendix,* Tables S4 and S5. The geothermobarometers employed in the estimation of P-T conditions in this study include the garnet-clinopyroxene Fe-Mg exchange geothermometer (GC) (4), garnet-clinopyroxene Fe-Mg geothermometer (GCK) (5), garnet-clinopyroxene-plagioclase-quartz geobarometer (GCPQ) (6), garnet-clinopyroxene geobarometer (GCB) (7) and the REE-based garnet-clinopyroxene thermobarometer (GC-REE) (8).

The GC geothermometer was recalibrated by Ravna (4) on the basis of a large number of previous experimental data, taking into account the relationship between X_{Ca}, Mg# and X_{Mn} of garnet and $\ln K_D$ with co-existing clinopyroxene, and is the most widely used garnet-clinopyroxene geothermometer available for a widest range of pressures (P) and temperatures (T), compositions, and can get the most accurate reproduction of experimental conditions. The applicable P-T range of this thermometer is P=1~8 GPa, T=600~1500°C (4). This thermometer can be employed on rocks with X_{Na}^{Cpx} less than 0.51 in clinopyroxene (jadeite component less than 0.51), otherwise the obtained temperatures will have large uncertainties. This is probably due to the fact that when the jadeite content in clinopyroxene is high, the system thermodynamic properties change and the corresponding activity model is altered (9), thus affecting the Fe^{2+}-Mg exchange in the system. The GCK geothermometer was recalibrated by Krogh (5) by fitting the quadratic linear relationship between X_{Ca} and $\ln K_D$ in garnet based on several experimental results (10–12). The GCK thermometer has basically the same principle as that of the GC thermometer, and has a small error (mostly within ± 50°C) in the calculated temperature conditions below 900°C, while in the conditions of 900–1500°C, the calculated temperature does not match well with the experimental temperature (13). The GCK geothermeter can be used on rocks with low jadeite composition in clinopyroxene (less than 0.44) (5), and is thus suitable for the temperature calculations

of the Shangying garnet clinopyroxenite.

The GCPQ geobarometer was recalibrated by Eckert et al. (6) for the co-existing garnet-plagioclase-plagioclase-quartz assemblages in granulite with small standard errors (less than ± ~5 kbar) (6, 14), thus has been widely used on the computation of pressures of granulite (such as the HP granulite in the literature of H. Y. C. Wang et al. 15–16). The GCB geobarometer is the latest barometer for co-existing garnet-clinopyroxene mineral pairs, which was calibrated by Beyer et al. (7) based on a series of piston-cylinder and multi-anvil experiments. The starting material for these experiments were hydrous and anhydrous synthetic mixtures of basaltic bulk compositions that yielded homogeneous bimineralic garnet–clinopyroxene assemblages. The GCB geobarometer is calibrated based on the pressure dependence of Ca-Tschermaks substitution in clinopyroxene, and can be applied on rocks metamorphosed at a wide range of P-T conditions between 2–7 GPa/700–1550°C with a narrow standard error of ± 4 kbar (7), making it the most accurate version at present. The GCB geobarometer is constructed for the mafic system, and is applicable to mantle eclogite with high-Na clinopyroxene, or garnet clinopyroxene with low-Na clinopyroxene, which is the case for rocks reported in this study (Na in Cpx=0.81-0.88 p.f.u). In the computation, we don't need activities of either jadeite or acmite components in clinopyroxene, thus the errors of chemical compositions of low-Na clinopyroxene (with low jadeite component) do not transfer to larger pressure errors in applying the geobarometers.

The GC-REE geothermometer is calibrated based on the exchange of rare earth elements (REEs) between garnet and clinopyroxene (8), in comparison to the typical garnet-clinopyroxene thermometer based on the Fe^{2+}-Mg exchange in the system. In general, trivalent REEs have a lower diffusion rate than that of divalent Fe and Mg (17), thus the GC-REE geothermometer can record higher closure temperatures of mafic-ultramafic rocks. Some practical examples of applying the GC-REE geothermometer and other traditional thermometers to calculate the temperatures of natural samples show that the retrieved temperatures calculated by the GC-REE geothermometer are in excellent agreement with those calculated by the widely used GCK geothermometer (5), verifying the accuracy of this thermometer. The GC-REE thermobarometer is developed for eclogite, granulite and garnet peridotites, and has been successfully employed for the calculation of the P-T conditions of the eight granulite xenoliths from Siberia (18), six granulites from granulite blocks in Central Finland (19), and four garnet pyroxenites from the Beni Bousera massif in Morocco (20), demonstrating that this thermometer can be used to calculate the metamorphic temperature and pressure conditions of the Shangying garnet clinopyroxenite with a low jadeite component in clinopyroxene.

Through detailed investigation under microscope and EPMA, the prograde metamorphism (M1) can be

represented by mineral assemblage including clinopyroxene (Cpx_1), plagioclase (Pl_1), and quartz (Qtz_1) enclosed in garnet (Grt_1). The metamorphic P-T conditions of the M1 phase were determined by the GC geothermometer (4), coupled with GCPQ geobarometer (6), which yield P-T conditions of 668–758°C/11.90–12.98 kbar. Since the Fe-Mg exchange thermometers are considered to be easily reset by diffusion, the net-transfer reactions and computed temperatures based on the REE exchange may be more accurate (8, 21–22). Thus, the GC-REE thermobarometer has been employed on two M1-phase Grt-Cpx pairs (*SI Appendix,* Table S5), which yield the 730 ± 20°C/11.8 ± 0.2 kbar and 706 ± 41°C/11.3 ± 0.4 kbar, respectively. In addition, the GCK (5) has been used, due to its excellent agreement in temperatures with the REE-in Grt-Cpx thermobarometer for the 35 well-equilibrated mantle eclogites evaluated by Sun and Liang (8), which yield temperatures of 642–749°C.

The peak metamorphic (M2) minerals include the core of garnet (Grt_2) and clinopyroxene (Cpx_2) associated with rutile. The metamorphic peak conditions were estimated to be 792–890°C/19.80–24.50 kbar by applying the GC geothermometer in concert with the GCB (7). The GC-REE thermobarometer employed on two Grt-Cpx mineral pairs (*SI Appendix,* Table S5) gives the peak metamorphic conditions of 844 ± 16°C/22.3 ± 0.2 kbar and 875 ± 38°C/21.2 ± 0.4 kbar, respectively. The GCK thermometer was further utilized, which yielded temperatures of 796–893°C, showing a good reproducibility comparable to the above estimation.

The retrograde mineral (M3) assemblage consists of the rims of garnet (Grt_3) and clinopyroxene (Cpx_3) associated with small plagioclase (Pl_3) and quartz grains (Qtz_3) around garnet. In the same way as the prograde phase, the metamorphic P-T conditions for the retrograde phase were retrieved to be 720–763°C/11.67–12.55 kbar by applying the GC geothermometer combined with GCPQ geobarometer. The GC-REE thermobarometer (*SI Appendix,* Table S5) yield the 749 ± 23°C/12.7 ± 0.3 kbar and 719 ± 28°C/10.7 ± 0.3 kbar, while the GCK thermometer yields temperatures of 685–740°C.

From the above M1, M2, and M3 assemblages and P-T calculations, we obtain three points along the P-T path, indicating a clockwise trend with a sharp, hairpin-like turn through the M2 calculated peak (Fig. 4). At present, the limited mineral assemblages prohibit construction of pseudosections that could further constrain the paths between the M1-M2-M3 stages. Thus we are presently unable to determine intermediate changes along this calibrated path. Future work aimed at acquiring samples with more varied mineral assemblages may yield a more complete metamorphic history.

The standard errors of these employed geothermobarometers are considered in the P-T computation (see the dashed line extending each P-T box of Fig. 3). In general, the reliability of the widely used Grt-Cpx Fe-Mg

thermometers is overestimated, and their standard error cited as ± 30 to 50°C is probably too optimistic (13, 23), in that the oxidation state of iron in clinopyroxene (i.e., Fe^{3+}/Fe^{Total}) is difficult to estimate accurately. In this work, we take ± 60°C as the uncertainties for the GCK and GC thermometers, according to results given in the literature (23). As for the geobarometers used in this study, the maximum estimate of uncertainties for the GCPQ geobarometer is ± ~5 kbar (6, 14). The GCB geobarometer is considered to be the most accurate Grt-Cpx barometer available at present, with a standard error of approximately ± 4 kbar (7). The plot in Fig. 3 can help to visually identify outliers in a dataset and determine the dispersion degree and bias in a dataset (i.e., via the length of the box, the shape of the upper and lower compartments, and the length of the solid line segments of the error bar). About 50% of the data is concentrated in the box and about 99.3% within the solid line segments of the error bar, thus the box plots can effectively help make estimates of the degree of dispersion of the dataset, and allow effective screening of temperature or pressure data retrieved by different geothermobarometers in this work. In the P-T dataset we obtained, the P-T plots reflect the most reliable range of temperature and pressure for each metamorphic stage, which we calculate as 668–758°C/11.3–13.0 kbar for M1, 792–890°C/19.8–24.5 kbar for M2, and 685–763°C/10.7–12.7 kbar for M3.

Zircon U-Pb geochronology and Hf isotopic composition

Zircons grains from metagabbros (18SY19C-3 and 19SY03-1) and a granitic dike (18SY-28) cutting through the metagabbro were separated and analyzed (*SI Appendix,* Tables S2 and S3). Zircon crystals in sampled metagabbro (18SY-19C-3) are relatively few, but most show anhedral, ellipsoidal shapes. Zircons are about 50–120 μm in length with aspect ratios of 1:1–1:2. Cathodoluminescence (CL) images reveal that most zircons exhibit core-mantle-rim texture, and the cores have low luminescence with a faintly broad platy zoning, whereas the rims show bright or relatively dark luminescence with no zoning (*SI Appendix,* Fig. S6*A*). Some grains with incomplete zoning and complex internal texture may indicate incomplete recrystallization, which is also evidenced by their age. All zircons are characterized by depleted LREE, negative Eu and positive Ce anomalies. The evaluation of common Pb is essential before obtaining accurate U-Pb dating results, since the presence of common lead contamination in zircons is a potentially serious source of error. The common Pb in all zircons of this study, calculated by the correction algorithm of Andersen (24), is negligible (mostly lower than 1 ppm), thus exerts a small effect on the age results. According to the CL images and U-Pb age, the zircons can be classified into four groups (*SI Appendix,* Fig. S6*A*): (1) Five concordant spots (red circles) on the dark cores have high Th (11.2–112 ppm), U (65–1,991.4 ppm) contents and variable Th/U (0.05–0.37) ratios. These

zircons are used to construct a reliable discordant line with an upper intercept age of 2,550 ± 16 Ma (MSWD = 0.47), and yield a weighted mean $^{207}Pb/^{206}Pb$ age of 2,528 ± 30 Ma (MSWD = 0.57), which, within the analytical error of the upper intercept age, is interpreted to represent the crystallization age of metagabbro. (2) The relatively dark rims (blue circles) show variable contents of Th (9.4–53.6 ppm), U (46.7–1,260.9 ppm) and low Th/U (0.04–0.2) ratios. Two analytical spots located on these areas yield a weighted mean $^{207}Pb/^{206}Pb$ age of 2,471 ± 38 Ma (MSWD = 0.36), which is considered as the age of metamorphism. (3) Four spots (orange circles) are analyzed on the incomplete recrystallization zircons, which have variable Th (32.6–114.1 ppm), U (170.5–313.4) contents and Th/U (0.15–0.43) ratios, and yield variable ages between 2,276–2,367 Ma. These ages may have no geological meaning since they are incompletely recrystallized, resulting in their estimated ages ranging between the original formation and the later thermal events (25). (4) the remaining two spots (green circles) located on the outmost bright rim with no zoning, which is a typical characteristic of metamorphic zircon (25–27). These zircons have low contents of Th (9.0–16.9 ppm), U (38.7–54.6 ppm) and Th/U (0.23–0.31) ratios, which show a weighted mean age of 1,823 ± 56 Ma (MSWD = 0.42), representing a much later stage of metamorphism.

Zircons from another metagabbro sample (19SY03-1) were analyzed to further supplement our data set. Most zircon grains are light brown to brown, columnar or anhedral in shape, with grain sizes varying between 50 × 100 μm and 100 × 200 μm. The core-mantle-rim texture revealed by the CL images is common (*SI Appendix,* Fig. S6*B*). Similar to the metagabbro described above, these zircons can be divided into four groups based on CL images. However, outermost bright rims are too thin to analyze, making it difficult to get their age. The characteristics of the other three groups of zircons are as follows (*SI Appendix,* Fig. S6*B*): (1) Twenty analytical spots (red circles in *SI Appendix,* Fig. S6*B*) are located on the inner core, which show low luminescence with weak zoning. These zircons have variable Th (4.9–232.1 ppm), U (53.4–3,249.2 ppm) contents and Th/U (0.01–1.23) ratios. These twenty spots show apparent $^{207}Pb/^{206}Pb$ ages between 2,498 and 2,598 Ma, yielding an upper intercept age of 2,523 ± 14 Ma (MSWD = 0.63) and a weighted mean age of 2,522 ± 15 Ma (MSWD = 0.45). (2) Nine zircons located on the mantle (blue circles) show relatively bright luminescence with fir-tree zoning, which have large variations of Th (26.8–165.5 ppm), U (33.9–278.9 ppm) concentrations with Th/U ratios of 0.21–1.25. These spots yield a weighted mean age of 2,474 ± 26 Ma (MSWD = 0.40). (3) Group 3 includes only one zircon (orange circles), which shows characteristics of metasomatic zircons with incomplete zoning or blurred internal zoning. This zircon has Th and U content of 57.9 ppm and 240.43 ppm with Th/U ratio of 0.24, which has $^{207}Pb/^{206}Pb$ age of 2,287 ± 35 Ma, which, as above, is a

geologically meaningless mixture age.

The Lu-Hf isotopic compositions of the metagabbro (18SY19C-3 and 19SY03-1) are presented in *SI Appendix,* Fig. S6*D* and Table S3. All $^{176}Lu/^{177}Hf$ ratios in zircons within two samples range from 0.000009 to 0.0012, suggesting that there is no obvious radiogenic Hf accumulation in these zircons after their formation. Therefore, the $^{176}Lu/^{177}Hf$ ratios determined in this study represent the Hf isotopic composition of the system at the time of zircon formation (28). For 18SY19C-3, all analyses of Lu-Hf isotopes conducted on the dated zircons yield a narrow range of $^{176}Hf/^{177}Hf$ ratios from 0.281335 to 0.281429. Five spots on the group 1 zircon show ε_{Hf} (t = 2,528 Ma) values between + 5.5 and + 7.7, with T_{DM1} values varying from 2,542 to 2,624 Ma. Two analyses on group 2, three analyses on group 3 and one spot on group 4 dated zircon show ε_{Hf} (t is the weighted mean $^{207}Pb/^{206}Pb$ age of each group or their respective $^{207}Pb/^{206}Pb$ age) values ranging from + 5.1 to + 6.9, + 4.3 to + 5.6, and – 6.1 respectively, corresponding to T_{DM1} values ranging from 2,517 to 2,585 Ma, 2,483 to 2,526 Ma, and 2,453 Ma.

For 19SY03-1, eleven Lu-Hf isotopic analytical spots conducted on the dated group 1 zircons yield $^{176}Hf/^{177}Hf$ ratios ranging from 0.281286 to 0.281383, and ε_{Hf} (t = 2,522 Ma) values between + 3.6 and + 6.9 (*SI Appendix,* Fig. S6*D*), with T_{DM1} values varying from 2,567 to 2,692 Ma.

A granitic dike (18SY-28) was dated to constrain the lower limit of the crystallization age of metagabbros. Zircons in this sample exhibit anhedral or prismatic shapes, and their length is about 100–170 μm with aspect ratios of 1:1–1:2. Most zircons show board or patched zoning texture under the CL images (*SI Appendix,* Fig. S6*C*). These zircons have strongly depleted LREE, positive Ce, and negative Eu anomalies. Thorium and U concentrations in twenty-three zircons range from 64 to 395.1 ppm, and 73.7 to 416.9 ppm, respectively, resulting in moderately high Th/U (0.65–1.80) ratios. Twenty-three analytical spots (red circles) yield an upper intercept age of 2,487 ± 15 Ma (MSWD = 0.35) and eleven most concordant spots yield a weighted mean $^{207}Pb/^{206}Pb$ age of 2,468 ± 23 Ma (MSWD = 0.23) (*SI Appendix,* Fig. S6*C*), which is identical to the upper intercept age within analytical error.

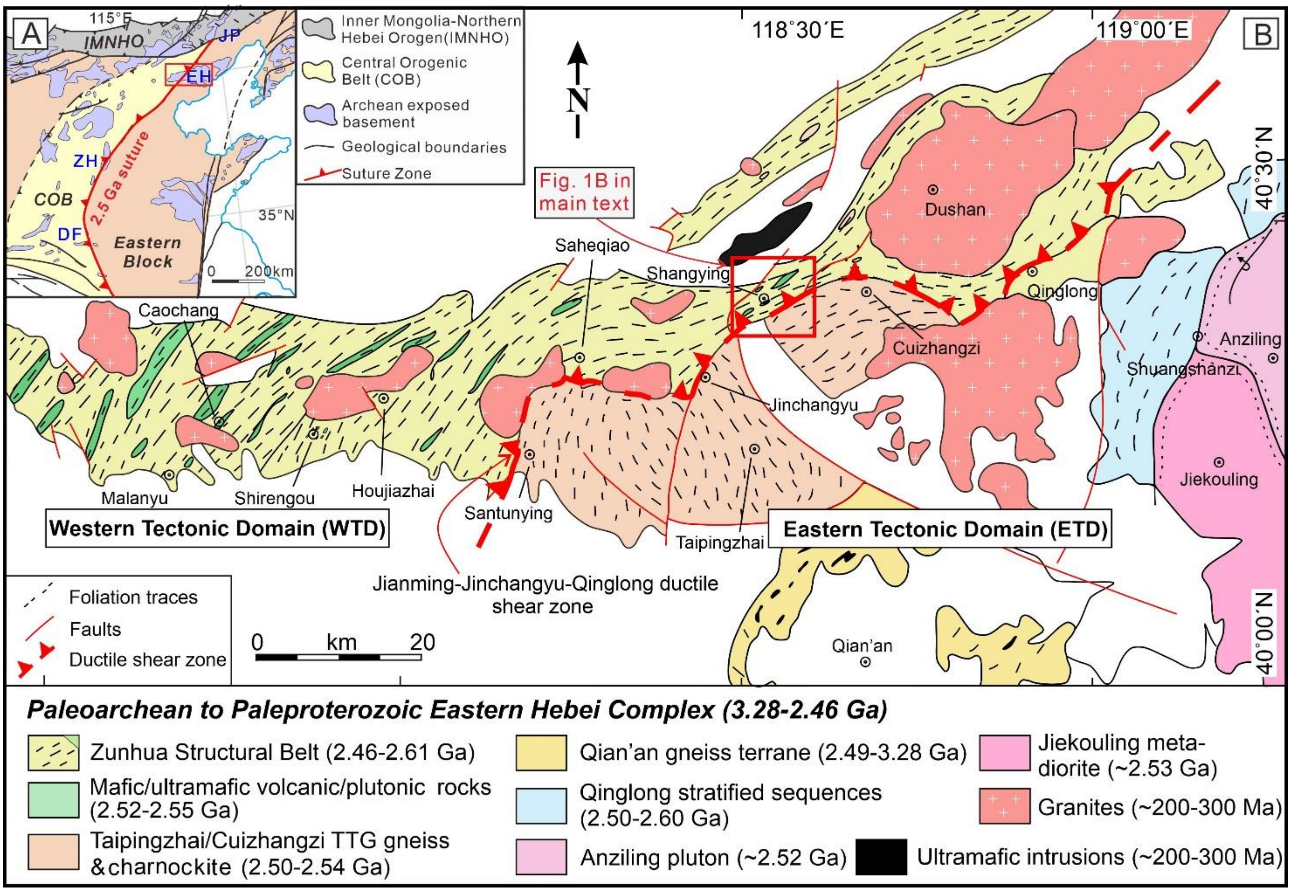

Fig. S1. (*A*) Simplified tectonic map of the COB showing the locations of the Jianping (JP), Eastern Hebei (EH), Zanhuang (ZH), and Dengfeng (DF) Complexes along the eastern margin of the COB. (*B*) Geological map of the Eastern Hebei Complex (29) showing the location of Shangying rock units.

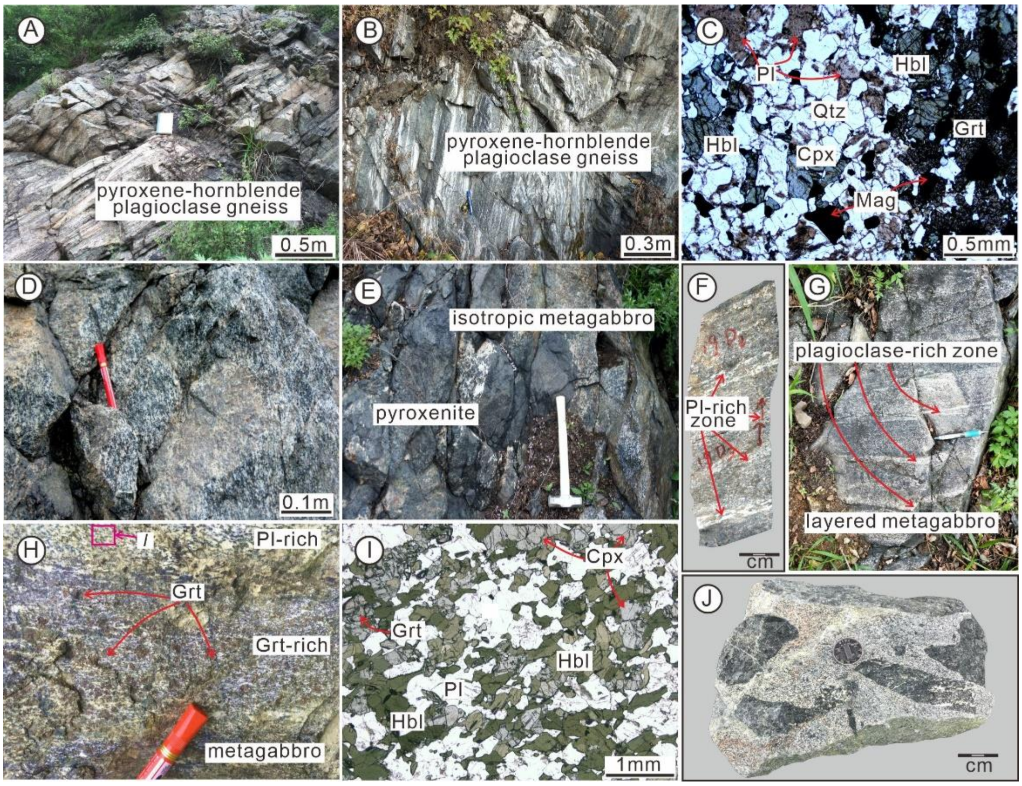

Fig. S2. (*A*–*C*) Field photos and photomicrographs of the pyroxene-hornblende plagioclase gneiss. (*D*) Field photos of the isotropic metagabbro. (*E*) Field photo of the pyroxenite enclosed in metagabbro. Note the distinct boundary between these two lithologies. (*F*–*G*) Field photos of layered metagabbro, containing layers of plagioclase-rich and garnet-rich assemblages. (*H*) Close-up field photograph of layered metagabbro. (*I*) Photomicrograph of the plagioclase-rich zone within layered metagabbro. (*J*) Hand specimen photo of small pyroxenite xenoliths in metagabbro.

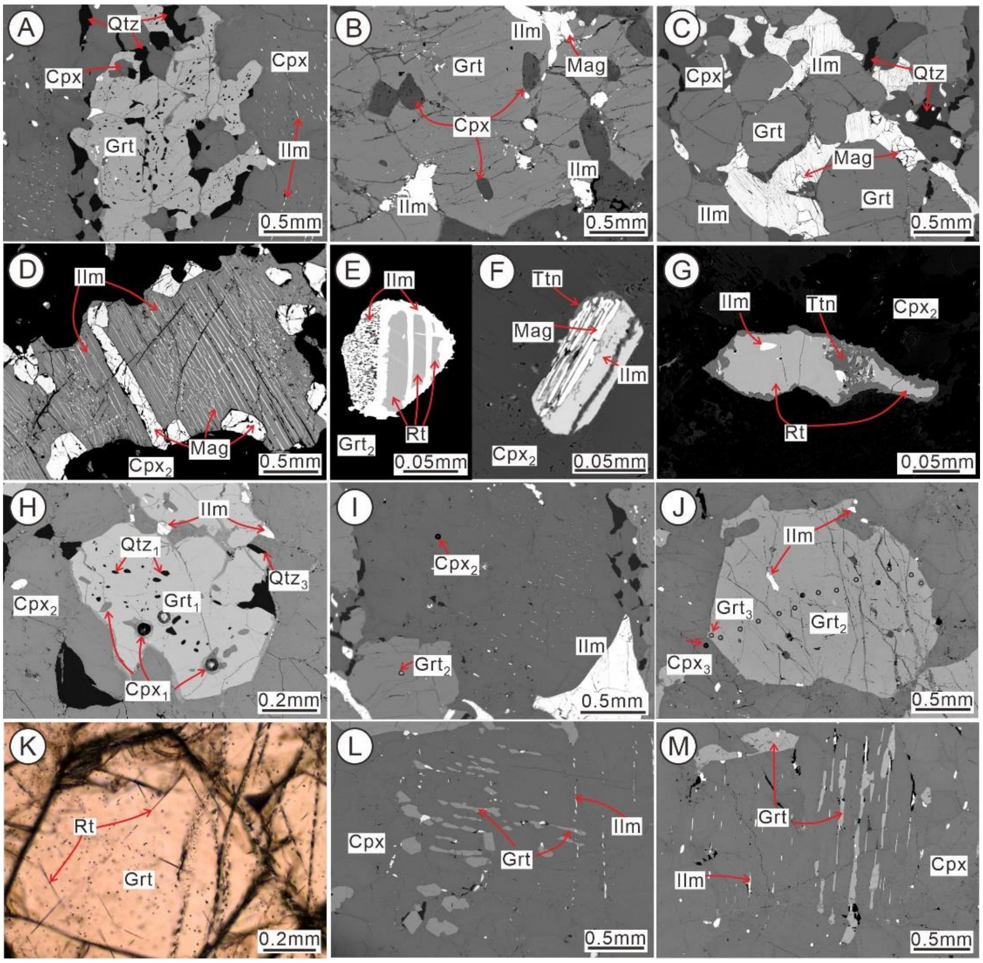

Fig. S3. (*A*) Extremely anhedral garnet with irregular grain boundaries, which might have been severely reformed during later metasomatism, and were thus avoided for P-T calculations. (*B*-*C*) Ilmenites (Ilm) intergrown with magnetite (Mag) appear in the gaps between the granular garnet (Grt) crystals, suggesting that they are the products of later stage metamorphism. (*D*) Large ilmenite crystals intergrown with magnetite. (*E*) Textures showing rutile inclusion within Grt_2 retrograded to ilmenite. (*F*) Euhedral Ilmenite intergrown with magnetite surrounded by titanite appear as inclusion in garnet. Note that the idiomorphic hexagonal ilmenite within garnet is separated by Al-rich phases in the center, which may indicate extremely high-pressure or high-temperature conditions (30). (*G*) Rutile inclusion within Cpx_2 retrograded to ilmenite and titanite. (*H*) Garnet with clinopyroxene (Cpx_1) and quartz inclusions (Qtz_1) surrounded by fine grains of quartz (Qtz_3) and ilmenite. The clinopyroxene (Cpx_1) and the garnet in close proximity to it (Grt1) were sampled to calculate the temperature and pressure conditions of M1 metamorphic stage. The sampling vestiges (round holes) by Laser-ablation-ICP-MS with a spot size of 44 μm for the REE P-T calculations of the prograde garnet and clinopyroxene are shown. (*I*) The cores of large and clean clinopyroxene (Cpx_2) and garnet (Grt_2) were sampled to calculate the temperature and pressure conditions of M2 metamorphic stage. (*J*) Trace element compositional profile path of a M2-M3 garnet were analyzed, which corresponds to the profile in *SI Appendix,*

Fig. S4*E*. We sampled the clinopyroxene rim (Cpx_3) close to the rim of the garnet (Grt_3), constituting a mineral pair for P-T computation (M3 metamorphic stage) applying the REE-based garnet-clinopyroxene thermobarometer. (*K*) Rutile (Rt) lamellae exsolved from within the garnet. (*L*–*M*) Aligned thin garnet rods exsolved from within the clinopyroxene.

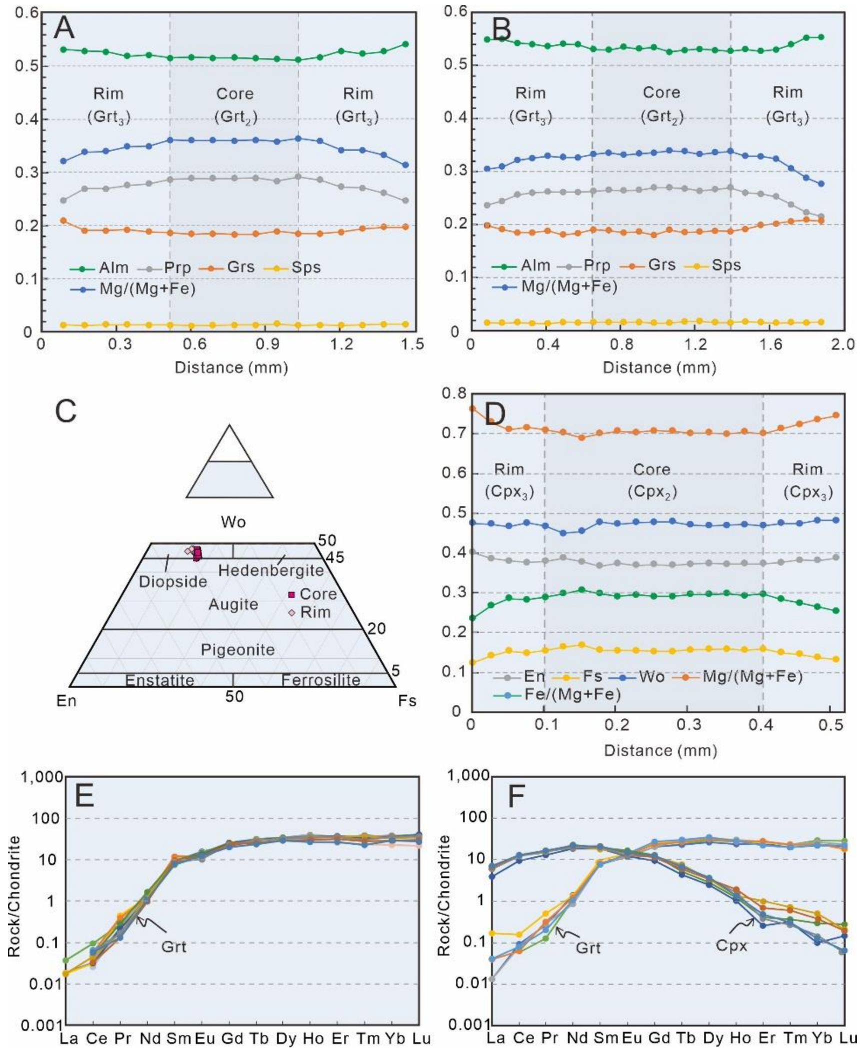

Fig. S4. Major and trace element characteristics of garnet and clinopyroxene in the Shangying garnet clinopyroxenite. (*A–B*) Compositional profiles of two garnet porphyroblasts (with rare inclusions) showing the zoning texture of garnet. Note the garnet core and rim refer to Grt_2 and Grt_3, respectively. (*C–D*) Classification and compositional profile of the clinopyroxene. (*E*) Chondrite-normalized REE profiles of garnet (Grt_2-Grt_3) in garnet clinopyroxenite, showing insignificant variation in REE from the core to rim. (*F*) Chondrite-normalized REE patterns for garnet (Grt_1-Grt_3) and clinopyroxene (Cpx_1-Cpx_3) used for P-T computation applying the REE-based garnet-clinopyroxene thermobarometer (*SI Appendix,* Table S5). The chondrite values are from Sun and McDonough (31).

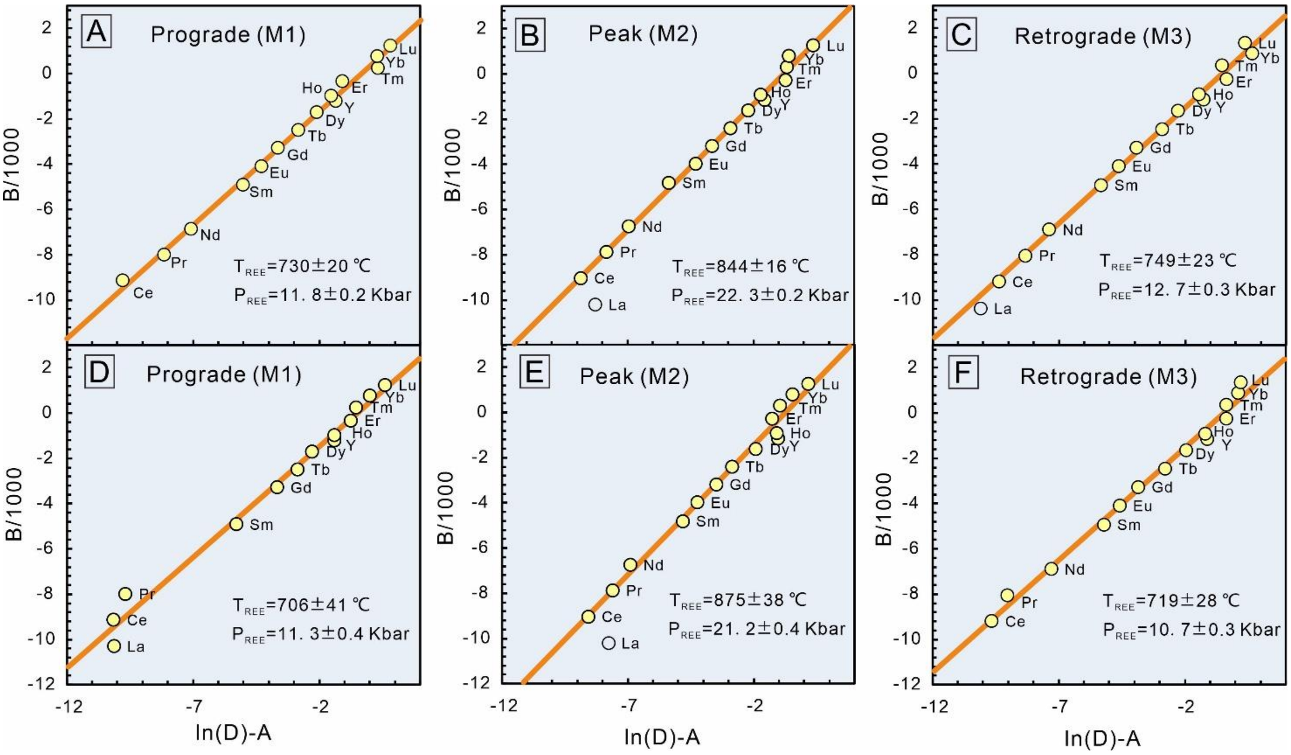

Fig. S5. Inversion plots for the different metamorphic phases of garnet clinopyroxenite applying the Grt-Cpx REE-based thermobarometer. Thick orange lines are obtained through least squares fits to filtered REE data (solid circles) after excluding the outliers (empty circles).

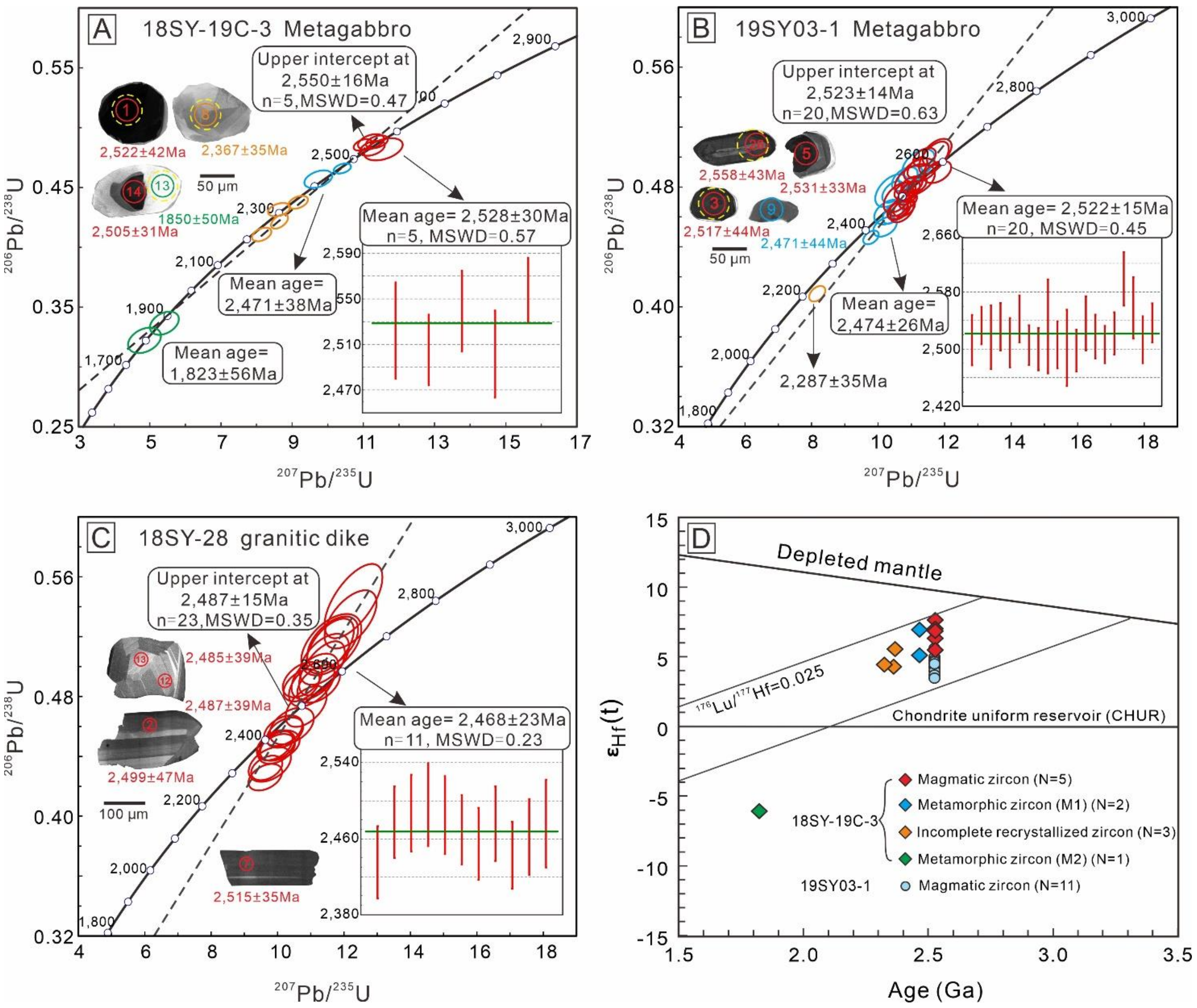

Fig. S6. Zircon U-Pb ages and Lu-Hf isotopic compositions from the metagabbros and granitic dike in the Shangying rock units. (*A*–*B*) Zircon cathodoluminescence (CL) images and concordia plots of two metagabbros. According to the CL images and trace element characteristics of zircons, ages can be divided into four groups: magmatic (red), first stage metamorphosed–ME-1 (blue), incomplete recrystallized (orange) and second stage metamorphosed–ME-2 (green). (*C*) Zircon CL images and concordia plots of granitic dike crosscutting the internal layering of the metagabbro, which can provide the lower limit of deformation and metamorphic age of the metagabbro. (*D*) Zircon Lu-Hf isotopic compositions of metagabbros. Hf isotope evolution lines with a $^{176}Lu/^{177}Hf$ ratio of 0.025 for the oceanic crust are drawn. The CL images for representative zircons from these samples are shown and the weighted mean age plots are inserted in the corresponding concordia plots. MSWD—mean square of weighted deviates.

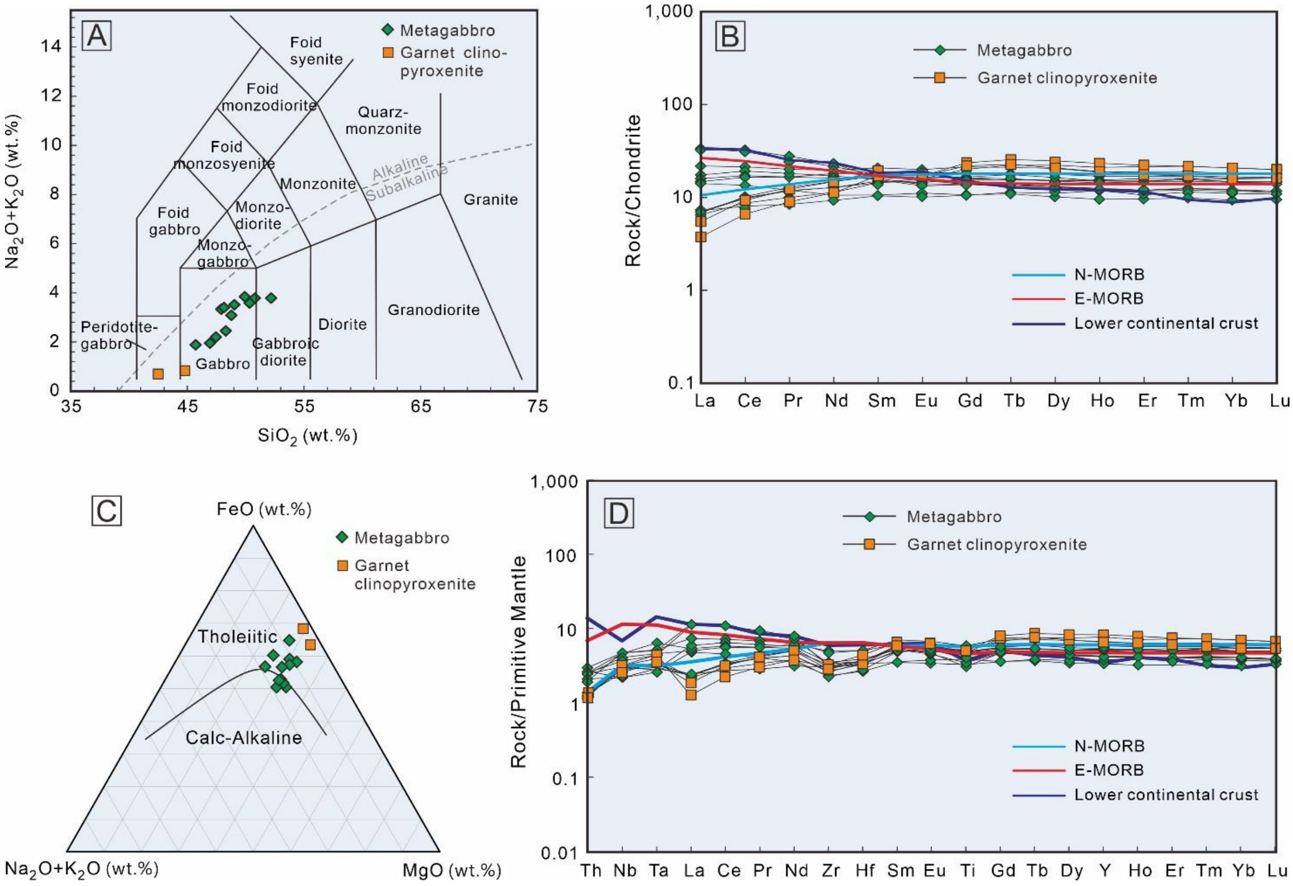

Fig. S7. Whole-rock compositions of metagabbro and garnet clinopyroxenite in the Shangying rock units. (*A*) Na_2O + K_2O versus SiO_2 geochemical classification diagram (32). (*B*) Chondrite-normalized trace element diagrams for metagabbro and garnet clinopyroxenite. (*C*) FeO_T-total alkalis (Na_2O + K_2O)-MgO diagram (33). (*D*) Primitive mantle-normalized trace element diagrams for metagabbro and garnet clinopyroxenite. The N-MORB (Normal Mid-Ocean Ridge Basalt), E-MORB (enriched Mid-Ocean Ridge Basalt), Lower continental crust and normalization values from Sun and McDonough (31).

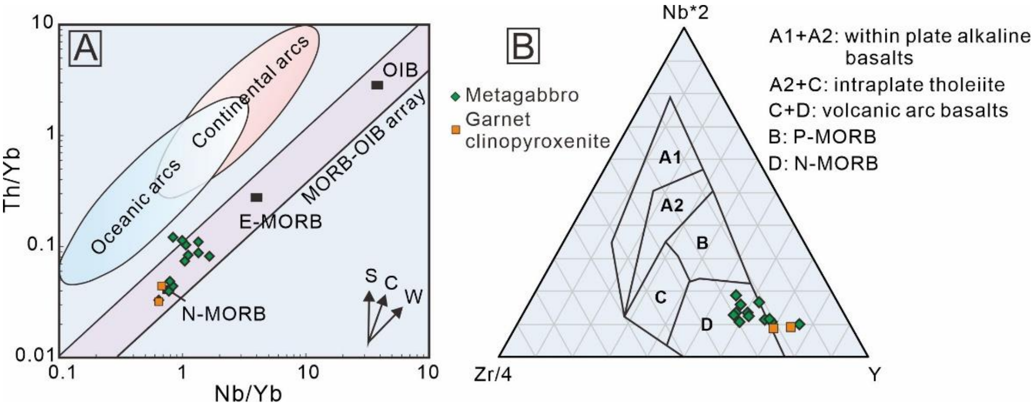

Fig. S8. Tectonic discrimination diagrams for metagabbro and garnet pyroxenite in the Shangying rock units. (*A*) Th/Yb versus Nb/Yb diagram adopted from Pearce (34). (*B*) Zr-Nb-Y diagram employed from Meschede (35). N-MORB—normal mid-ocean ridge basalt; E-MORB—enriched mid-ocean ridge basalt; OIB—ocean-island basalt; P-MORB—plume-influenced mid-ocean ridge basalt; S—subduction component vector; C—crustal contamination vector; W—within-plate variation.

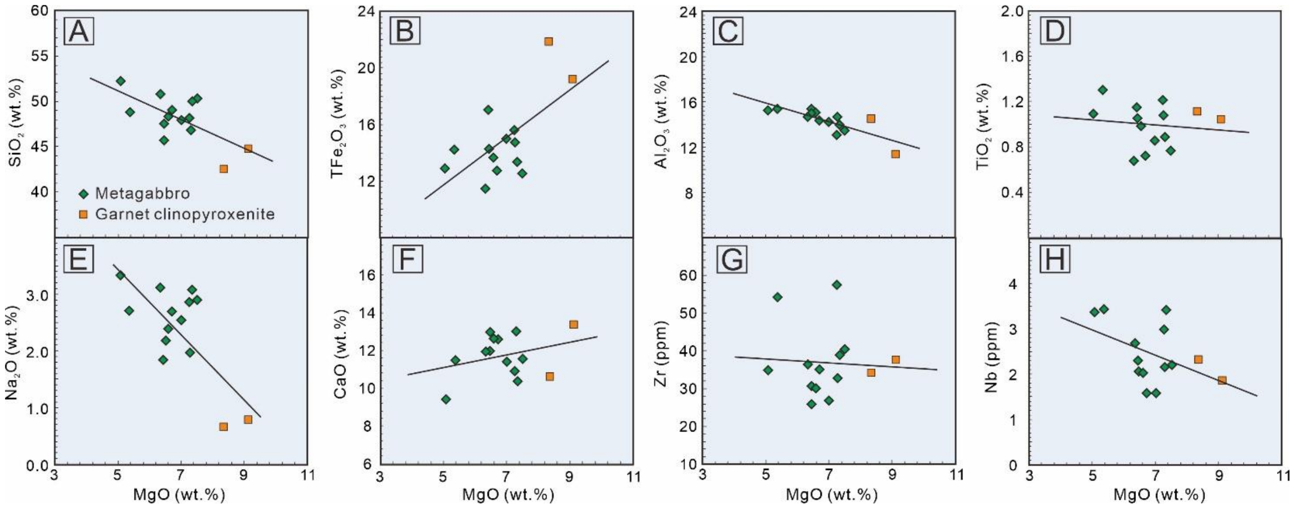

Fig. S9. Variation diagrams of MgO versus several major and trace elements for metagabbros and garnet clinopyroxenites in the Shangying rock units. Data are provided in *SI Appendix,* Table S1. Note the metagabbros and garnet clinopyroxenites share the same compositional trends, showing they have a close genetic relationship.

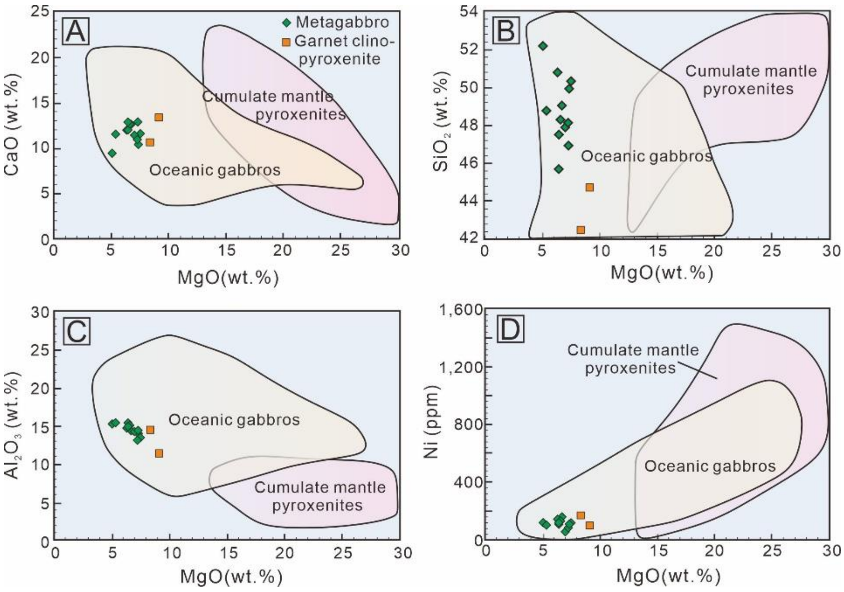

Fig. S10. Bulk major elements plots for the Shangying garnet clinopyroxenites compared with oceanic gabbros and mantle-derived cumulate pyroxenite (36, 37). The Shangying garnet clinopyroxenites are distinct from mantle cumulate pyroxenite by showing significantly lower MgO and higher Al_2O_3 contents, but they are similar to oceanic gabbro.

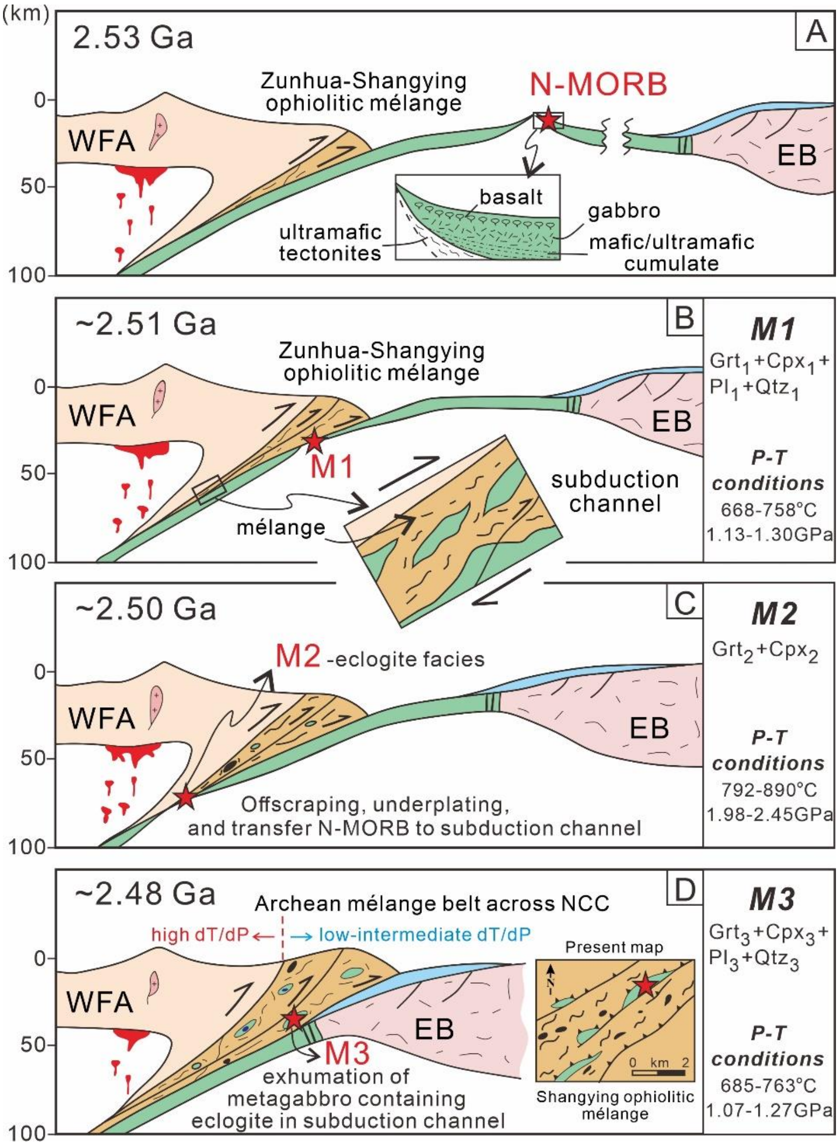

Fig. S11. Schematic model for the formation and exhumation of the Shangying eclogite-facies garnet clinopyroxenite during late Archean to early Proterozoic. The main tectonic events leading to the development of the map pattern, successive metamorphic mineral assemblages, and P-T conditions of three main metamorphic stages experienced by the Shangying garnet clinopyroxenite are shown. (*A*–*B*) The newborn oceanic crust with N-MORB geochemical affinity formed at the mid-ocean ridge, and was subducted to ~37–43 km, undergoing prograde granulite-facies metamorphism (M1), perhaps related to ridge subduction. The N-MORB is off-scraped and incorporated into the subduction channel. (*C*) When subduction reaches 65–70 km, eclogite-facies metamorphism (M2) occurred, and then the eclogite-facies oceanic slices are decoupled from the descending oceanic lithosphere, transferred to the subduction channel, and were extruded by the upward flow in the subduction channel, where they were structurally fragmented into isolated blocks forming components of the Zunhua-Shangying ophiolitic mélange (*D*). The paired metamorphic belt relationships are extrapolated from the along-strike equivalents in the Dengfeng Complex (38) that are consistent with P-T

conditions from Eastern Hebei (39). Insert in panel D is a simplified version of our geological map presented in Fig. 2 in the main text. When the metagabbro blocks containing eclogite were exhumed to ~35–42 km, they underwent retrograde metamorphism (M3). Abbreviations in the figure: WFA–Wutai/Fuping arc terrane; EB–Eastern Block.

Table S1. Major (wt%) and trace element (ppm) compositions of the metagabbros and garnet clinopyroxenites in the Shangying rock units of the Zunhua-Shangying ophiolitic mélange, North China Craton.

	Metagabbro												Garnet clinopyroxenite	
	19SY03-1	19SY03-2	19SY03-3	19SY04-1	19SY56-1	19SY56-2	19SY58-1	19SY58-2	19SY71-1	19SY89-1	18SY19C-3	18SY19C-4	18SY19C-1	18SY19C-2
SiO_2	49.81	48.95	50.79	49.98	51.46	48.44	45.74	48.08	47.88	47.40	47.35	46.78	44.97	42.69
TiO_2	0.89	0.72	0.68	0.76	1.08	1.29	1.15	0.98	0.86	1.19	1.05	1.08	1.05	1.12
Al_2O_3	13.94	14.33	14.68	13.35	15.02	15.28	15.38	14.98	14.22	12.91	15.09	14.61	11.45	14.52
$Fe_2O_3^T$	13.36	12.74	11.49	12.48	12.75	14.14	17.07	13.62	15.00	15.38	14.25	14.69	19.28	21.93
MgO	7.32	6.70	6.34	7.45	5.00	5.33	6.45	6.56	7.00	7.14	6.43	7.26	9.17	8.39
CaO	10.35	12.57	11.96	11.50	9.28	11.43	11.99	12.59	11.41	10.76	12.93	12.97	13.42	10.68
Na_2O	3.07	2.69	3.11	2.88	3.29	2.68	1.84	2.38	2.54	2.82	2.19	1.96	0.78	0.65
K_2O	0.77	0.82	0.67	0.68	0.45	0.39	0.05	0.06	0.80	0.53	0.05	0.05	0.04	0.05
P_2O_5	0.06	0.06	0.08	0.06	0.09	0.11	0.11	0.08	0.04	0.12	0.09	0.09	0.08	0.06
MnO	0.18	0.23	0.21	0.18	0.19	0.23	0.31	0.20	0.22	0.22	0.21	0.19	0.30	0.35
LOI	0.56	0.52	0.39	1.01	1.01	0.64	0.23	0.76	0.24	1.13	0.65	0.49	0.11	0.25
Total	100.30	100.34	100.38	100.32	99.62	99.95	100.30	100.29	100.19	99.60	100.30	100.16	100.64	100.69
Mg#	52.05	51.01	52.21	54.20	43.72	42.75	42.80	48.81	48.04	47.93	47.21	49.48	48.52	43.13
Cr	191	246	310	191	193	203	257	219	6.91	151	226	240	229	253
Co	63.9	81.7	58.0	52.3	79.7	60.8	71.5	69.5	75.8	60.3	66.5	73.5	87.9	115
Ni	111	161	146	120	122	105	109	133	57.8	86.3	124	124	102	172
Cu	8.30	130	102	61.9	164	119	132	101	156	42.2	129	64.9	28.0	238
Zn	88.4	81.9	80.2	79.9	106	103	84.4	93.1	93.8	120	87.3	99.1	113	105
Pb	3.21	1.92	2.53	3.79	1.31	1.60	0.25	0.44	2.21	1.43	0.35	0.32	0.38	0.21
Rb	3.25	10.2	4.05	5.70	7.46	6.29	0.14	0.18	10.5	5.55	0.17	0.13	0.17	0.17
Sr	213	171	187	176	245	311	111	156	259	164	159	148	41.3	39.7
Ba	291	147	147	107	108	115	8.50	13.1	85.3	142	11.5	8.43	9.68	5.63

V	297	294	265	282	334	360	347	328	382	366	334	353	397	380
Nb	3.42	1.58	2.68	2.20	3.37	3.43	2.30	2.03	1.58	2.98	2.06	2.16	1.86	2.32
Ta	0.21	0.12	0.18	0.12	0.27	0.22	0.17	0.14	0.11	0.19	0.12	0.14	0.15	0.19
Zr	38.7	34.8	36.3	40.2	34.7	54.0	25.7	29.9	26.6	57.3	30.5	32.6	37.4	34.0
Hf	1.13	1.06	1.16	1.23	1.15	1.61	0.87	1.07	0.84	1.60	1.04	1.14	1.38	1.04
Th	0.17	0.23	0.22	0.22	0.22	0.26	0.11	0.12	0.18	0.21	0.11	0.11	0.11	0.10
U	0.04	0.07	0.06	0.04	0.01	0.07	0.00	0.01	0.02	0.05	0.01	0.01	0.01	0.01
Y	19.9	17.5	19.3	19.5	25.2	31.2	35.8	23.8	15.6	27.1	24.8	24.1	30.5	38.4
La	7.97	1.71	7.67	4.15	3.77	5.15	1.54	1.68	3.36	3.52	1.64	1.61	1.30	0.89
Ce	19.0	4.92	19.8	11.7	10.5	13.0	5.31	6.10	8.27	10.1	5.94	6.24	5.71	4.04
Pr	2.41	0.80	2.64	1.72	1.58	1.92	0.95	1.13	1.14	1.57	1.12	1.22	1.17	0.86
Nd	10.1	4.34	10.8	7.95	7.56	9.15	5.29	5.97	4.92	8.44	5.96	6.91	6.93	5.24
Sm	2.66	1.59	2.46	2.37	2.44	3.19	2.21	2.20	1.62	2.78	2.30	2.60	2.97	2.64
Eu	0.88	0.58	0.80	0.77	0.95	1.16	0.93	0.88	0.64	1.08	0.84	0.94	0.98	0.99
Gd	2.84	2.17	2.86	2.82	3.40	4.39	4.13	3.16	2.19	3.76	3.29	3.36	4.52	4.85
Tb	0.52	0.42	0.49	0.50	0.66	0.79	0.83	0.58	0.41	0.68	0.58	0.59	0.85	0.95
Dy	3.32	2.80	3.26	3.33	4.15	5.20	5.64	3.82	2.58	4.50	3.89	3.87	5.28	6.27
Ho	0.69	0.67	0.69	0.71	0.85	1.06	1.19	0.81	0.54	0.96	0.87	0.86	1.07	1.32
Er	1.99	1.76	1.94	1.99	2.48	3.14	3.55	2.45	1.59	2.79	2.64	2.49	2.92	3.69
Tm	0.32	0.29	0.30	0.31	0.41	0.48	0.55	0.38	0.25	0.43	0.41	0.39	0.43	0.55
Yb	2.06	1.88	1.99	2.06	2.48	3.08	3.53	2.49	1.59	2.84	2.70	2.52	2.73	3.53
Lu	0.30	0.28	0.29	0.29	0.36	0.44	0.50	0.36	0.24	0.42	0.41	0.38	0.41	0.51
(La/Yb)cn	2.77	0.65	2.77	1.45	1.09	1.20	0.31	0.48	1.52	0.89	0.44	0.46	0.34	0.18
(La/Sm)cn	1.93	0.69	2.01	1.13	1.00	1.04	0.45	0.49	1.34	0.82	0.46	0.40	0.28	0.22
(Gd/Yb)cn	1.14	0.96	1.19	1.14	1.13	1.18	0.97	1.05	1.14	1.10	1.01	1.10	1.37	1.14
(Eu/Eu*)cn	0.97	0.96	0.92	0.91	1.01	0.95	0.93	1.02	1.04	1.02	0.93	0.98	0.82	0.84
(Ce/Ce*)cn	1.05	1.03	1.08	1.08	1.06	1.02	1.05	1.05	1.03	1.05	1.04	1.04	1.05	1.03
Al_2O_3/TiO	15.66	19.84	21.59	17.49	13.93	11.81	13.37	15.25	16.63	10.81	14.31	13.52	10.93	12.99

2														
Zr/Hf	34.17	32.83	31.29	32.65	30.22	33.56	29.37	27.86	31.77	35.83	29.26	28.67	27.03	32.53
Nb/Ta	16.21	13.10	14.69	18.91	12.58	15.84	13.94	14.38	14.66	15.55	16.59	15.68	12.55	12.43
Zr/Y	1.95	2.00	1.88	2.06	1.38	1.73	0.72	1.25	1.71	2.12	1.23	1.35	1.22	0.88
Nb/Nb*	1.34	0.78	1.07	1.15	1.04	1.16	1.22	1.18	0.93	1.17	1.34	1.29	1.05	1.13
Zr/Zr*	0.62	0.94	0.55	0.73	0.67	0.81	0.68	0.68	0.75	0.90	0.70	0.66	0.70	0.84
Hf/Hf*	0.78	1.03	0.85	0.89	0.87	0.87	0.78	0.91	0.90	0.91	0.85	0.84	0.89	0.75
Ti/Ti*	0.82	0.94	0.66	0.76	0.89	0.84	0.85	0.86	1.05	0.88	0.93	0.89	0.72	0.73
North	40°23'25.5″	40°23'25.5″	40°23'25.5″	40°23'24.0″	40°23'56.6″	40°23'56.6″	40°24'5.1″	40°24'5.1″	40°21'49.8″	40°20'36.6″	40°24'5.1″	40°24'5.1″	40°24'5.1″	40°24'5.1″
East	118°28'25.4″	118°28'25.4″	118°28'25.4″	118°28'22.5″	118°32'9.0″	118°32'9.0″	118°32'24.5″	118°32'24.5″	118°29'49.8″	118°28'32.0″	118°32'24.0″	118°32'24.0″	118°32'24.0″	118°32'24.0″

$Fe_2O_3^T$: total iron as Fe_2O_3; Mg# = Mg / (Mg + Fe^{2+}); cn: chondrite normalized; Eu/Eu*, Ce/Ce*, Nb/Nb*, Zr/Zr* and Ti/Ti* ratios following the method of Taylor and McLennan (40).

Table S2. LA-ICP-MS U-Pb analytical data for zircons of the metagabbros and granitic dike in the Shangying rock units of the Zunhua-Shangying ophiolitic mélange, North China Craton.

Spot number	Types of zircon	Pb (ppm)	Th (ppm)	U (ppm)	Common Pb (ppm)	Th/U	$^{207}Pb/^{206}Pb$	1σ	$^{207}Pb/^{235}U$	1σ	$^{206}Pb/^{238}U$	1σ	$^{208}Pb/^{232}Th$	1σ	$^{207}Pb/^{206}Pb$	1σ	$^{207}Pb/^{235}U$	1σ	$^{206}Pb/^{238}U$	1σ
**Sample 18SY19C-3 Metagabbro (N40°24'5.1″, E118°32′24.0″)**																				
18SY19C-3-01	MA	910.07	112.00	1,741.63	0	0.06	0.1654	0.0040	11.2145	0.2399	0.4881	0.0043	0.1269	0.0032	2,522	42	2,541	20	2,562	19
18SY19C-3-02	ME-1	32.86	9.36	46.72	0.61	0.20	0.1543	0.0056	9.8651	0.3924	0.4582	0.0073	0.1469	0.0095	2,464	28	2,462	37	2,481	32
18SY19C-3-03	IR	243.94	114.11	266.82	0.29	0.43	0.1509	0.0031	9.1699	0.1816	0.4377	0.0035	0.1181	0.0024	2,367	35	2,355	18	2,340	16
18SY19C-3-04	IR	246.13	108.19	313.36	0	0.35	0.1482	0.0029	8.6466	0.1574	0.4216	0.0040	0.1140	0.0022	2,325	34	2,301	17	2,268	18
18SY19C-3-05	IR	108.03	35.63	170.54	0	0.21	0.1439	0.0033	8.6326	0.2038	0.4315	0.0037	0.1155	0.0036	2,276	39	2,300	22	2,312	17
18SY19C-3-06	IR	113.34	32.58	206.50	0	0.16	0.1432	0.0033	8.1433	0.1805	0.4107	0.0038	0.1121	0.0036	2,333	39	2,247	20	2,218	17
18SY19C-3-07	ME-2	33.74	16.97	54.57	0.03	0.31	0.1098	0.0055	5.4106	0.2680	0.3366	0.0064	0.1025	0.0060	1,850	50	1,887	42	1,966	30
18SY19C-3-08	MA	344.77	57.05	563.59	0	0.10	0.1647	0.0030	11.3809	0.2030	0.4881	0.0035	0.1419	0.0036	2,505	31	2,555	17	2,606	15
18SY19C-3-09	ME-1	612.23	53.61	1,260.89	0.07	0.04	0.1611	0.0026	10.4108	0.1599	0.4659	0.0028	0.1306	0.0032	2,478	27	2,472	14	2,466	12
18SY19C-3-10	MA	253.16	98.16	267.89	0.44	0.37	0.1682	0.0035	11.2513	0.2326	0.4829	0.0042	0.1315	0.0027	2,539	35	2,544	19	2,540	18
18SY19C-3-11	MA	73.31	18.37	96.39	0.05	0.19	0.1644	0.0066	11.5655	0.3558	0.4820	0.0061	0.1508	0.0149	2,502	38	2,570	29	2,536	27
18SY19C-3-12	MA	1,031.23	99.19	1,991.40	0.45	0.05	0.1631	0.0026	10.9854	0.1699	0.4856	0.0032	0.1320	0.0028	2,558	28	2,522	14	2,552	14
18SY19C-3-13	ME-2	18.34	9.01	38.75	0	0.23	0.1107	0.0072	4.8523	0.3052	0.3223	0.0070	0.0907	0.0060	1,811	35	1,794	53	1,801	34
**Sample 19SY03-1 Metagabbro (N40°23'25.5″, E118°28′25.42″)**																				
19SY03-1-01	MA	195.00	103.32	117.91	0.38	0.88	0.1655	0.0035	10.6365	0.2276	0.4651	0.0047	0.1285	0.0024	2,513	35	2,492	20	2,462	21
19SY03-1-02	MA	222.74	40.18	357.52	0.38	0.11	0.1675	0.0026	10.9068	0.1718	0.4707	0.0035	0.1270	0.0034	2,533	26	2,515	15	2,487	15
19SY03-1-03	MA	258.87	139.42	113.10	0	1.23	0.1658	0.0044	11.2688	0.3244	0.4911	0.0052	0.1387	0.0024	2,517	44	2,546	27	2,575	22
19SY03-1-04	ME-1	114.59	55.07	66.82	0.07	0.82	0.1639	0.0044	11.2164	0.3201	0.4953	0.0065	0.1409	0.0032	2,496	46	2,541	27	2,593	28
19SY03-1-05	MA	355.21	123.92	390.17	0	0.32	0.1673	0.0034	10.7868	0.2121	0.4660	0.0038	0.1328	0.0025	2,531	33	2,505	18	2,466	17
19SY03-1-06	MA	445.32	232.10	280.81	0	0.83	0.1651	0.0034	10.5899	0.2061	0.4644	0.0040	0.1298	0.0021	2,509	35	2,488	18	2,459	17
19SY03-1-07	MA	206.01	87.97	161.47	0	0.54	0.1684	0.0034	11.6760	0.2948	0.4994	0.0071	0.1355	0.0028	2,542	33	2,579	24	2,611	31

19SY03-1-08	ME-1	53.77	26.83	33.95	0.8	0.79	0.1591	0.0054	10.4006	0.3215	0.4775	0.0072	0.1298	0.0042	2,447	57	2,471	29	2,516	32
19SY03-1-09	ME-1	93.65	46.14	68.21	0.13	0.68	0.1615	0.0046	10.3404	0.3020	0.4633	0.0055	0.1281	0.0032	2,471	44	2,466	27	2,454	24
19SY03-1-10	MA	409.16	97.05	574.26	0.22	0.17	0.1647	0.0027	10.6228	0.1794	0.4663	0.0038	0.1444	0.0030	2,506	28	2,491	16	2,467	17
19SY03-1-11	MA	373.79	129.29	426.89	0	0.30	0.1632	0.0029	10.7682	0.1836	0.4775	0.0036	0.1270	0.0023	2,500	30	2,503	16	2,516	16
19SY03-1-12	MA	68.68	52.39	3,249.17	0.56	0.02	0.1674	0.0066	11.1087	0.3976	0.4847	0.0071	0.1342	0.0035	2,532	66	2,532	33	2,548	31
19SY03-1-13	MA	345.17	160.11	256.86	0.26	0.62	0.1648	0.0032	11.3975	0.2225	0.4999	0.0040	0.1285	0.0023	2,506	33	2,556	18	2,613	17
19SY03-1-14	MA	85.84	44.84	53.42	0.26	0.84	0.1644	0.0051	10.6059	0.2893	0.4729	0.0066	0.1273	0.0030	2,502	53	2,489	25	2,496	29
19SY03-1-15	ME-1	283.64	165.46	132.85	0	1.25	0.1591	0.0034	10.4485	0.2138	0.4767	0.0047	0.1275	0.0021	2,446	37	2,475	19	2,513	21
19SY03-1-16	MA	306.78	161.74	156.36	0.44	1.03	0.1641	0.0028	11.1813	0.1951	0.4930	0.0046	0.1344	0.0021	2,498	29	2,538	16	2,584	20
19SY03-1-17	MA	134.12	69.65	80.39	0.63	0.87	0.1678	0.0038	10.7194	0.2307	0.4638	0.0049	0.1294	0.0023	2,536	38	2,499	20	2,456	21
19SY03-1-18	ME-1	157.84	83.92	87.04	0.19	0.96	0.1615	0.0039	10.8037	0.2562	0.4853	0.0052	0.1300	0.0024	2,472	46	2,506	22	2,550	23
19SY03-1-19	MA	349.76	94.04	460.24	0	0.20	0.1660	0.0030	10.8628	0.1903	0.4734	0.0035	0.1341	0.0025	2,518	31	2,511	16	2,498	15
19SY03-1-20	ME-1	1,477.52	34.22	40.85	0.08	0.84	0.1585	0.0025	9.7860	0.1549	0.4463	0.0030	0.1370	0.0032	2,440	33	2,415	15	2,379	14
19SY03-1-21	MA	700.20	176.89	952.35	0	0.19	0.1649	0.0025	10.8751	0.1670	0.4769	0.0037	0.1342	0.0020	2,506	26	2,513	14	2,514	16
19SY03-1-22	MA	179.29	41.00	258.52	0.53	0.16	0.1664	0.0029	10.9739	0.1900	0.4778	0.0043	0.1308	0.0036	2,522	30	2,521	16	2,517	19
19SY03-1-23	IR	162.33	57.90	240.43	0	0.24	0.1448	0.0029	8.1718	0.1602	0.4087	0.0034	0.1141	0.0028	2,287	35	2,250	18	2,209	16
19SY03-1-24	ME-1	209.11	57.94	278.96	0.1	0.21	0.1632	0.0028	10.4469	0.2040	0.4625	0.0046	0.1325	0.0030	2,500	29	2,475	18	2,451	20
19SY03-1-25	MA	102.94	39.17	98.74	0	0.40	0.1742	0.0040	11.7873	0.2722	0.4900	0.0049	0.1297	0.0034	2,598	38	2,588	22	2,571	21
19SY03-1-26	MA	171.47	90.02	92.01	0	0.98	0.1700	0.0043	11.7727	0.2868	0.5032	0.0059	0.1310	0.0029	2,558	43	2,586	23	2,627	25
19SY03-1-27	ME-1	217.69	96.18	163.04	0.37	0.59	0.1629	0.0037	10.9713	0.2401	0.4876	0.0042	0.1366	0.0026	2,487	38	2,521	20	2,560	18
19SY03-1-28	MA	272.01	76.45	337.72	0	0.23	0.1656	0.0032	11.0172	0.2093	0.4815	0.0036	0.1389	0.0027	2,513	33	2,525	18	2,534	16
19SY03-1-29	ME-1	261.91	149.90	123.33	0.1	1.22	0.1633	0.0039	10.2081	0.2341	0.4534	0.0043	0.1307	0.0021	2,490	41	2,454	21	2,411	19
19SY03-1-30	MA	176.59	4.93	350.73	0.58	0.01	0.1679	0.0027	11.1885	0.1796	0.4817	0.0035	0.1631	0.0097	2,537	27	2,539	15	2,534	15
**Sample 18SY-28**	**Granitic dike**	**(N40°24′5.10″, E118°32′24″)**																		
18SY-28-01	MA	558.53	271.51	409.57	0	0.66	0.1579	0.0035	10.7455	0.2407	0.4912	0.0048	0.1558	0.0039	2,435	38	2,501	21	2,576	21
18SY-28-02	MA	256.91	116.14	146.51	0	0.79	0.1642	0.0045	11.6592	0.3134	0.5160	0.0071	0.1835	0.0053	2,499	47	2,577	25	2,682	30
18SY-28-03	MA	133.43	63.96	73.66	1.51	0.87	0.1638	0.0050	11.6167	0.3698	0.5146	0.0084	0.1725	0.0052	2,496	52	2,574	30	2,676	36
18SY-28-04	MA	318.78	178.75	208.91	0.5	0.86	0.1621	0.0036	10.2225	0.2241	0.4559	0.0041	0.1389	0.0032	2,477	38	2,455	20	2,421	18
18SY-28-05	MA	355.29	170.73	243.98	0.6	0.70	0.1628	0.0039	11.1240	0.2869	0.4933	0.0063	0.1579	0.0046	2,487	40	2,534	24	2,585	27

18SY-28-06	MA	278.76	162.75	161.83	0.16	1.01	0.1639	0.0042	10.4261	0.2714	0.4603	0.0050	0.1384	0.0036	2,496	44	2,473	24	2,441	22
18SY-28-07	MA	701.12	395.05	416.85	0.25	0.95	0.1657	0.0035	10.8607	0.2522	0.4734	0.0056	0.1444	0.0035	2,515	35	2,511	22	2,498	24
18SY-28-08	MA	297.74	149.88	166.86	0.82	0.90	0.1664	0.0038	11.2399	0.2632	0.4886	0.0052	0.1590	0.0036	2,522	39	2,543	22	2,565	23
18SY-28-09	MA	360.25	120.93	185.67	0	0.65	0.1649	0.0048	12.2867	0.3559	0.5438	0.0101	0.2255	0.0073	2,506	48	2,627	27	2,799	42
18SY-28-10	MA	222.98	113.62	142.10	1.12	0.80	0.1651	0.0040	11.3360	0.2814	0.4988	0.0074	0.1545	0.0047	2,509	41	2,551	23	2,609	32
18SY-28-11	MA	277.79	119.93	158.28	0.32	0.76	0.1663	0.0041	12.1974	0.3204	0.5316	0.0079	0.1726	0.0047	2,521	42	2,620	25	2,748	33
18SY-28-12	MA	226.88	109.09	140.33	0.72	0.78	0.1630	0.0038	11.5842	0.2911	0.5138	0.0074	0.1632	0.0045	2,487	39	2,571	24	2,673	32
18SY-28-13	MA	224.56	112.63	126.64	0	0.89	0.1628	0.0038	11.4104	0.3019	0.5049	0.0070	0.1609	0.0043	2,485	39	2,557	25	2,635	30
18SY-28-14	MA	257.70	132.07	159.29	1.07	0.83	0.1628	0.0040	11.0260	0.2790	0.4884	0.0062	0.1476	0.0036	2,485	41	2,525	24	2,564	27
18SY-28-15	MA	253.45	156.39	145.11	1.45	1.08	0.1617	0.0044	9.7659	0.2598	0.4347	0.0047	0.1276	0.0029	2,473	45	2,413	25	2,327	21
18SY-28-16	MA	319.09	196.81	205.99	0	0.96	0.1628	0.0037	9.6309	0.2066	0.4261	0.0036	0.1244	0.0026	2,485	38	2,400	20	2,288	17
18SY-28-17	MA	531.23	378.09	241.93	0.41	1.56	0.1612	0.0033	9.6736	0.1928	0.4323	0.0034	0.1206	0.0020	2,468	35	2,404	18	2,316	15
18SY-28-18	MA	321.71	201.52	187.27	0	1.08	0.1613	0.0035	10.0012	0.2133	0.4469	0.0040	0.1286	0.0024	2,469	36	2,435	20	2,381	18
18SY-28-19	MA	244.80	148.53	184.26	0.11	0.81	0.1599	0.0036	9.9135	0.2206	0.4474	0.0041	0.1246	0.0024	2,455	38	2,427	21	2,384	18
18SY-28-20	MA	182.98	125.14	102.67	0.27	1.22	0.1618	0.0038	9.9739	0.2266	0.4450	0.0040	0.1224	0.0022	2,476	39	2,432	21	2,373	18
18SY-28-21	MA	378.92	200.58	252.43	0	0.79	0.1587	0.0033	10.7242	0.2227	0.4875	0.0044	0.1468	0.0028	2,443	35	2,500	19	2,560	19
18SY-28-22	MA	413.83	292.44	162.15	0	1.80	0.1606	0.0038	10.1706	0.2353	0.4571	0.0042	0.1292	0.0024	2,462	40	2,450	21	2,427	18
18SY-28-23	MA	285.56	177.85	175.85	0	1.01	0.1618	0.0044	10.0246	0.2621	0.4473	0.0041	0.1305	0.0028	2,476	46	2,437	24	2,383	18

Note: MA: Magmatic zircon in origin; ME-1: Metamorphic zircon in origin (Late Neoarchean-Early Paleoproterozoic metamorphism); ME-2: Metamorphic zircon in origin (Late Paleoproterozoic metamorphism); IR: Incomplete recrystallized zircon. The common Pb was calculated using Andersen's algorithm (24) with modelling of 3D discordance pattern without knowledge of ^{204}Pb. This method requires precise measurements of the ratios of ^{206}Pb/^{238}U, ^{207}Pb/^{235}U, ^{208}Pb/^{232}Th and ^{232}Th/^{238}U, with the knowledge of the composition of common Pb, which was developed as a last-resort approach to be applied in situations where ^{204}Pb cannot be determined with any degree of confidence, e.g., because of severe Hg contamination in LA-ICP-MS analysis.

Table S3. MC-LA-ICP-MS Lu-Hf isotopic data for zircons of the metagabbros in the Shangying rock units of the Zunhua-Shangying ophiolitic mélange, North China Craton.

Spot number	Types of zircon	T(Ma)	$^{176}Yb/^{177}Hf$	1σ	$^{176}Lu/^{177}Hf$	±1σ	$^{176}Hf/^{177}Hf$	±1σ	$\varepsilon_{Hf}(t)$	T_{DM1}^{Hf}(Ma)	T_{DM2}^{Hf}(Ma)
**Sample 18SY19C-3**	**Metagabbro**	**(N40°24'5.1″, E118°32′24.0″)**									
18SY19C-3-01	MA	2,528	0.00134013	3.51671E-05	4.39529E-05	1.27659E-06	0.2813485	1.20859E-05	6.4	2,592	2,632
18SY19C-3-02	ME-1	2,471	0.00039895	2.54135E-05	9.17453E-06	2.53435E-07	0.28140313	2.15828E-05	7.0	2,517	2,546
18SY19C-3-03	IR	2,361	0.0011191	2.80452E-05	2.15769E-05	3.55324E-07	0.28139703	2.40077E-05	4.3	2,526	2,630
18SY19C-3-04	IR	2,367	0.00099039	5.21629E-05	1.74972E-05	7.04927E-07	0.28142913	1.86692E-05	5.6	2,483	2,556
18SY19C-3-05	IR	2,325	0.00110253	2.46728E-05	2.30947E-05	3.03418E-07	0.28142489	1.03816E-05	4.4	2,489	2,592
18SY19C-3-07	ME-2	1,823	0.00166084	3.6569E-05	3.46162E-05	5.92059E-07	0.28145237	1.7478E-05	-6.1	2,453	2,851
18SY19C-3-08	MA	2,528	0.00354026	1.81863E-05	0.000116629	3.41112E-07	0.28138918	1.59336E-05	7.7	2,542	2,551
18SY19C-3-09	ME-1	2,471	0.00172507	1.34808E-05	5.47907E-05	2.43464E-07	0.28135416	1.64496E-05	5.2	2,585	2,657
18SY19C-3-10	MA	2,528	0.0036836	3.70922E-05	0.000133249	1.54159E-06	0.2813721	1.70489E-05	7.0	2,566	2,590
18SY19C-3-11	MA	2,528	0.00707784	1.5045E-05	0.00026696	3.2683E-07	0.28133525	1.53801E-05	5.5	2,624	2,684
18SY19C-3-13	MA	2,528	0.00318901	1.9766E-05	0.000100815	6.98566E-07	0.2813656	1.44275E-05	6.9	2,573	2,601
**Sample 19SY03-1**	**Metagabbro**	**(N40°23'25.5″, E118°28′25.42″)**									
19SY03-4-01	MA	2,522	0.01135588	0.000117178	0.00042717	3.29523E-06	0.28130539	1.70256E-05	4.0	2,675	2,769
19SY03-4-02	MA	2,522	0.0234306	0.000285771	0.00109225	1.13586E-05	0.2813831	2.29494E-05	5.7	2,615	2,670
19SY03-4-03	MA	2,522	0.01328083	4.09989E-05	0.00062004	1.62439E-06	0.2813299	2.04883E-05	4.6	2,655	2,736
19SY03-4-05	MA	2,522	0.0136773	0.00010727	0.00056337	4.0211E-06	0.28139244	1.97222E-05	6.9	2,567	2,595
19SY03-4-06	MA	2,522	0.00690841	3.37665E-05	0.00026634	2.466E-06	0.28129157	1.97526E-05	3.8	2,682	2,782
19SY03-4-07	MA	2,522	0.00763153	3.69611E-05	0.00029577	7.45804E-07	0.28134512	2.46799E-05	5.7	2,613	2,669
19SY03-4-10	MA	2,522	0.00721215	2.93181E-05	0.00027185	5.98892E-07	0.28130497	2.07433E-05	4.3	2,665	2,754
19SY03-4-11	MA	2,522	0.01593168	0.000142181	0.00072265	4.62549E-06	0.28134896	3.56444E-05	5.1	2,637	2,706
19SY03-4-13	MA	2,522	0.00841763	1.8901E-05	0.00036342	5.24039E-07	0.28132149	1.67455E-05	4.7	2,649	2,728
19SY03-4-16	MA	2,522	0.00807169	5.90847E-05	0.00030831	2.42027E-06	0.28128692	2.06759E-05	3.6	2,692	2,797

19SY03-4-17	MA	2,522	0.00940717	2.73986E-05	0.00036334	1.42839E-06	0.28131588	2.89991E-05	4.5	2,657	2,740
19SY03-4-19	MA	2,522	0.01135588	0.000117178	0.00042717	3.29523E-06	0.28130539	1.70256E-05	4.0	2,675	2,769
19SY03-4-21	MA	2,522	0.0234306	0.000285771	0.00109225	1.13586E-05	0.2813831	2.29494E-05	5.7	2,615	2,670
19SY03-4-22	MA	2,522	0.01328083	4.09989E-05	0.00062004	1.62439E-06	0.2813299	2.04883E-05	4.6	2,655	2,736
19SY03-4-23	IR	2,522	0.0136773	0.00010727	0.00056337	4.0211E-06	0.28139244	1.97222E-05	6.9	2,567	2,595

Note: MA: Magmatic zircon in origin; ME-1: Metamorphic zircon in origin (Late Neoarchean-Early Paleoproterozoic metamorphism); ME-2: Metamorphic zircon in origin (Late Paleoproterozoic metamorphism); IR: Incomplete recrystallized zircon.

Table S4. Representative major elements of garnet, clinopyroxene, plagioclase in the garnet clinopyroxenite from the Shangying rock units of the Zunhua-Shangying ophiolitic mélange, North China Craton.

	Garnet line1 (rim–core-rim)																			
	Grt01-1	Grt01-2	Grt01-3	Grt01-4	Grt01-5	Grt01-6	Grt01-7	Grt01-8	Grt01-9	Grt01-10	Grt01-11	Grt01-12	Grt01-13	Grt01-14	Grt01-15	Grt01-16	Grt01-17	Grt02-1	Grt02-2	Grt02-3
SiO_2	38.65	38.48	38.80	38.83	38.75	38.83	39.24	39.02	38.94	38.96	39.14	38.90	39.12	38.62	38.63	38.36	38.40	38.93	38.57	38.66
TiO_2	0.03	0.08	0.02	0.08	0.02	0.00	0.01	0.03	0.06	0.11	0.00	0.04	0.14	0.08	0.11	0.09	0.08	0.15	0.04	0.11
Al_2O_3	22.19	22.11	22.21	22.28	22.45	22.15	22.09	22.32	22.09	22.14	22.08	22.19	22.28	22.09	21.96	22.06	22.07	22.10	22.23	22.39
FeO	23.90	24.28	24.06	23.79	24.05	23.26	23.87	23.95	23.89	23.79	23.71	23.83	23.64	24.07	23.92	23.83	24.80	24.48	24.97	24.81
MnO	0.61	0.57	0.65	0.64	0.61	0.59	0.56	0.57	0.61	0.62	0.71	0.58	0.59	0.58	0.61	0.68	0.69	0.69	0.66	0.73
MgO	6.25	6.95	6.89	7.10	7.23	7.26	7.49	7.54	7.50	7.51	7.37	7.63	7.35	6.97	6.94	6.62	6.34	5.92	6.23	6.58
CaO	7.35	6.85	6.81	6.90	6.80	6.57	6.65	6.73	6.63	6.64	6.83	6.72	6.62	6.67	6.93	6.95	7.07	6.90	6.77	6.62
Na_2O	0.00	0.01	0.00	0.00	0.00	0.00	0.00	0.03	0.00	0.01	0.02	0.00	0.00	0.04	0.00	0.03	0.01	0.00	0.00	0.00
K_2O	0.00	0.00	0.00	0.00	0.00	0.01	0.00	0.00	0.01	0.00	0.02	0.01	0.01	0.00	0.00	0.00	0.00	0.00	0.00	0.00
Cr_2O_3	0.08	0.07	0.09	0.14	0.06	0.08	0.10	0.07	0.05	0.06	0.10	0.05	0.08	0.10	0.04	0.12	0.08	0.10	0.03	0.07
Total	99.06	99.38	99.55	99.75	99.97	98.75	100.01	100.26	99.78	99.83	99.98	99.95	99.84	99.23	99.15	98.74	99.53	99.26	99.50	99.96
O	12.00	12.00	12.00	12.00	12.00	12.00	12.00	12.00	12.00	12.00	12.00	12.00	12.00	12.00	12.00	12.00	12.00	12.00	12.00	12.00
Si	3.00	2.99	3.00	2.99	2.98	3.01	3.01	2.99	3.00	3.00	3.01	2.99	3.01	3.00	3.00	2.99	2.99	3.02	3.00	2.99
Ti	0.00	0.00	0.00	0.00	0.00	0.00	0.00	0.00	0.00	0.01	0.00	0.01	0.01	0.00	0.01	0.01	0.00	0.01	0.00	0.01
Al	2.03	2.02	2.02	2.02	2.04	2.02	2.00	2.02	2.01	2.01	2.00	2.01	2.02	2.02	2.01	2.03	2.02	2.02	2.04	2.04
Fe^{3+}	0.00	0.00	0.00	0.00	0.00	0.00	0.00	0.00	0.00	0.00	0.00	0.00	0.00	0.00	0.00	0.00	0.00	0.00	0.00	0.00
Fe^{2+}	1.55	1.58	1.56	1.53	1.55	1.51	1.53	1.54	1.54	1.53	1.52	1.53	1.52	1.56	1.55	1.56	1.61	1.59	1.62	1.60
Mn	0.04	0.04	0.04	0.04	0.04	0.04	0.04	0.04	0.04	0.04	0.05	0.04	0.04	0.04	0.04	0.04	0.05	0.05	0.04	0.05
Mg	0.72	0.80	0.79	0.82	0.83	0.84	0.86	0.86	0.86	0.86	0.84	0.87	0.84	0.81	0.80	0.77	0.74	0.69	0.72	0.76
Ca	0.61	0.57	0.56	0.57	0.56	0.55	0.55	0.55	0.55	0.55	0.56	0.55	0.54	0.55	0.58	0.58	0.59	0.57	0.56	0.55
Na	0.00	0.00	0.00	0.00	0.00	0.00	0.00	0.00	0.00	0.00	0.00	0.00	0.00	0.00	0.00	0.00	0.00	0.00	0.00	0.00
K	0.00	0.00	0.00	0.00	0.00	0.00	0.00	0.00	0.00	0.00	0.00	0.00	0.00	0.00	0.00	0.00	0.00	0.00	0.00	0.00

Cr	0.00	0.00	0.01	0.01	0.00	0.00	0.01	0.00	0.00	0.00	0.01	0.00	0.00	0.01	0.00	0.01	0.00	0.01	0.00	0.00
Sum	7.98	8.00	7.99	7.99	8.00	7.97	7.99	8.00	8.00	8.00	7.99	8.00	7.98	7.99	7.99	7.99	8.00	7.96	7.99	7.99
**Mg#**	0.32	0.34	0.34	0.35	0.35	0.36	0.36	0.36	0.36	0.36	0.36	0.36	0.36	0.34	0.34	0.33	0.31	0.30	0.31	0.32
**Alm**	0.53	0.53	0.53	0.52	0.52	0.51	0.52	0.51	0.52	0.51	0.51	0.51	0.52	0.53	0.52	0.53	0.54	0.55	0.55	0.54
**Gross**	0.21	0.19	0.19	0.19	0.19	0.19	0.18	0.18	0.18	0.18	0.19	0.18	0.18	0.19	0.19	0.20	0.20	0.20	0.19	0.19
**Pyp**	0.25	0.27	0.27	0.28	0.28	0.29	0.29	0.29	0.29	0.29	0.28	0.29	0.29	0.27	0.27	0.26	0.25	0.24	0.24	0.26
**Spess**	0.01	0.01	0.01	0.01	0.01	0.01	0.01	0.01	0.01	0.01	0.02	0.01	0.01	0.01	0.01	0.02	0.02	0.02	0.01	0.02

Garnet line2 (rim–core-rim)

	Grt02-4	Grt02-5	Grt02-6	Grt02-7	Grt02-8	Grt02-9	Grt02-10	Grt02-11	Grt02-12	Grt02-13	Grt02-14	Grt02-15	Grt02-16	Grt02-17	Grt02-18	Grt02-19	Grt02-20	Grt02-21	Grt02-22	Grt02-23
SiO_2	38.48	38.69	38.65	38.67	38.70	38.57	38.78	38.67	38.63	38.32	38.81	38.84	38.71	38.40	38.73	38.47	38.90	38.37	38.52	38.24
TiO_2	0.11	0.06	0.03	0.06	0.04	0.04	0.07	0.03	0.04	0.10	0.03	0.03	0.06	0.10	0.04	0.04	0.00	0.03	0.04	0.09
Al_2O_3	22.23	22.36	22.15	22.15	22.02	22.03	22.19	22.16	22.24	21.95	22.01	22.07	22.31	22.08	22.25	22.22	21.93	21.87	21.83	22.03
FeO	24.83	24.36	24.65	24.76	24.06	23.95	24.52	24.33	24.62	23.95	23.97	24.45	24.10	24.20	24.49	24.20	24.41	24.63	25.08	25.86
MnO	0.66	0.62	0.75	0.69	0.71	0.72	0.71	0.76	0.68	0.66	0.79	0.83	0.73	0.70	0.80	0.73	0.68	0.71	0.69	0.75
MgO	6.72	6.67	6.69	6.73	6.69	6.73	6.80	6.81	6.98	6.89	6.81	6.82	6.81	6.95	6.74	6.65	6.54	6.09	5.68	5.56
CaO	6.65	6.68	6.44	6.58	6.74	6.67	6.62	6.67	6.49	6.77	6.55	6.71	6.69	6.71	6.92	7.12	7.27	7.36	7.41	7.45
Na_2O	0.00	0.03	0.00	0.02	0.02	0.02	0.00	0.02	0.00	0.02	0.00	0.00	0.00	0.00	0.02	0.01	0.00	0.00	0.01	0.01
K_2O	0.00	0.00	0.00	0.02	0.00	0.01	0.01	0.00	0.01	0.00	0.00	0.01	0.01	0.00	0.00	0.00	0.01	0.01	0.01	0.00
Cr_2O_3	0.02	0.00	0.07	0.02	0.04	0.07	0.06	0.04	0.02	0.01	0.02	0.03	0.07	0.00	0.04	0.05	0.07	0.04	0.05	0.06
Total	99.70	99.46	99.44	99.67	99.00	98.81	99.77	99.48	99.71	98.67	98.99	99.78	99.48	99.13	100.02	99.49	99.82	99.11	99.31	100.04
O	12.00	12.00	12.00	12.00	12.00	12.00	12.00	12.00	12.00	12.00	12.00	12.00	12.00	12.00	12.00	12.00	12.00	12.00	12.00	12.00
Si	2.98	3.00	3.00	2.99	3.01	3.00	3.00	3.00	2.99	2.99	3.02	3.00	3.00	2.99	2.99	2.98	3.01	3.00	3.01	2.98
Ti	0.01	0.00	0.00	0.00	0.00	0.00	0.00	0.00	0.00	0.01	0.00	0.00	0.00	0.01	0.00	0.00	0.00	0.00	0.00	0.01
Al	2.03	2.04	2.02	2.02	2.02	2.02	2.02	2.02	2.03	2.02	2.02	2.01	2.03	2.02	2.02	2.03	2.00	2.01	2.01	2.02
Fe^{3+}	0.00	0.00	0.00	0.00	0.00	0.00	0.00	0.00	0.00	0.00	0.00	0.00	0.00	0.00	0.00	0.00	0.00	0.00	0.00	0.00
Fe^{2+}	1.61	1.58	1.60	1.60	1.56	1.56	1.58	1.58	1.59	1.56	1.56	1.58	1.56	1.57	1.58	1.57	1.58	1.61	1.64	1.68
Mn	0.04	0.04	0.05	0.05	0.05	0.05	0.05	0.05	0.04	0.04	0.05	0.05	0.05	0.05	0.05	0.05	0.04	0.05	0.05	0.05

Mg	0.78	0.77	0.77	0.78	0.78	0.78	0.78	0.79	0.80	0.80	0.79	0.79	0.79	0.81	0.78	0.77	0.75	0.71	0.66	0.65
Ca	0.55	0.55	0.54	0.55	0.56	0.56	0.55	0.55	0.54	0.57	0.55	0.56	0.55	0.56	0.57	0.59	0.60	0.62	0.62	0.62
Na	0.00	0.00	0.00	0.00	0.00	0.00	0.00	0.00	0.00	0.00	0.00	0.00	0.00	0.00	0.00	0.00	0.00	0.00	0.00	0.00
K	0.00	0.00	0.00	0.00	0.00	0.00	0.00	0.00	0.00	0.00	0.00	0.00	0.00	0.00	0.00	0.00	0.00	0.00	0.00	0.00
Cr	0.00	0.00	0.00	0.00	0.00	0.00	0.00	0.00	0.00	0.00	0.00	0.00	0.00	0.00	0.00	0.00	0.00	0.00	0.00	0.00
Sum	8.00	7.98	7.99	7.99	7.98	7.98	7.99	7.99	8.00	8.00	7.98	7.99	7.99	8.00	8.00	8.00	7.99	8.00	7.99	8.01
**Mg#**	0.33	0.33	0.33	0.33	0.33	0.33	0.33	0.33	0.34	0.34	0.34	0.33	0.34	0.34	0.33	0.33	0.32	0.31	0.29	0.28
**Alm**	0.54	0.54	0.54	0.54	0.53	0.53	0.53	0.53	0.53	0.53	0.53	0.53	0.53	0.53	0.53	0.53	0.53	0.54	0.55	0.56
**Gross**	0.19	0.19	0.18	0.18	0.19	0.19	0.19	0.19	0.18	0.19	0.19	0.19	0.19	0.19	0.19	0.20	0.20	0.21	0.21	0.21
**Pyp**	0.26	0.26	0.26	0.26	0.26	0.27	0.26	0.27	0.27	0.27	0.27	0.26	0.27	0.27	0.26	0.26	0.25	0.24	0.22	0.22
**Spess**	0.01	0.01	0.02	0.02	0.02	0.02	0.02	0.02	0.01	0.01	0.02	0.02	0.02	0.02	0.02	0.02	0.02	0.02	0.02	0.02

	Garnet Core near inclusions (Prograde phase-**M1**)						Garnet Core (Peak metamorphic phase-**M2**)													
	Grt- I - 1	Grt- I- 2	Grt- I - 3	Grt- I - 4	Grt- I - 5	Grt- I - 6	Grt-C-1	Grt-C-2	Grt-C-3	Grt-C-4	Grt-C-5	Grt-C-6	Grt-C-7	Grt-C-8	Grt-C-9	Grt-C-10	Grt-C-11	Grt-R-1	Grt-R-2	Grt-R-3
SiO_2	38.83	38.62	38.96	38.80	38.91	38.82	39.21	38.95	38.94	39.22	39.17	39.00	39.06	38.69	39.11	38.61	39.00	38.42	38.66	38.67
TiO_2	0.06	0.00	0.03	0.10	0.04	0.05	0.00	0.00	0.03	0.12	0.07	0.03	0.09	0.00	0.03	0.08	0.04	0.06	0.08	0.01
Al_2O_3	22.19	22.33	22.75	22.35	22.37	22.40	22.46	21.66	22.13	22.53	22.43	22.48	22.84	22.34	22.24	22.26	22.34	22.23	22.15	22.10
FeO	23.31	22.80	24.06	23.75	24.46	23.68	23.60	23.67	23.10	24.18	23.64	23.37	23.86	23.28	23.12	23.50	23.53	24.34	24.27	24.34
MnO	0.64	0.66	0.66	0.71	0.84	0.70	0.60	0.66	0.72	0.53	0.53	0.67	0.58	0.63	0.52	0.59	0.60	0.64	0.70	0.81
MgO	7.26	7.01	7.00	6.15	6.66	6.82	6.88	6.09	6.48	6.95	6.91	7.18	6.85	6.53	7.01	6.66	6.75	6.68	6.86	6.74
CaO	6.92	7.15	7.26	7.21	6.80	7.07	6.88	7.76	7.35	7.34	7.16	7.23	7.28	7.66	7.26	7.64	7.35	6.72	6.78	6.96
Na_2O	0.03	0.02	0.05	0.05	0.02	0.03	0.00	0.00	0.02	0.01	0.02	0.01	0.00	0.02	0.03	0.02	0.01	0.00	0.01	0.00
K_2O	0.01	0.01	0.00	0.01	0.00	0.01	0.01	0.00	0.00	0.01	0.00	0.00	0.02	0.00	0.00	0.00	0.00	0.01	0.00	0.00
Cr_2O_3	0.00	0.00	0.05	0.06	0.00	0.02	0.05	0.02	0.00	0.04	0.02	0.00	0.00	0.03	0.00	0.00	0.02	0.00	0.08	0.02
Total	99.24	98.60	100.81	99.17	100.09	99.58	99.68	98.81	98.76	100.93	99.94	99.96	100.58	99.18	99.32	99.36	99.65	99.10	99.56	99.64
O	12.00	12.00	12.00	12.00	12.00	12.00	12.00	12.00	12.00	12.00	12.00	12.00	12.00	12.00	12.00	12.00	12.00	12.00	12.00	12.00
Si	3.00	3.00	2.97	3.01	3.00	3.00	3.01	3.04	3.02	2.99	3.01	2.99	2.98	3.00	3.02	2.99	3.01	2.99	2.99	2.99
Ti	0.00	0.00	0.00	0.01	0.00	0.00	0.00	0.00	0.00	0.01	0.00	0.00	0.01	0.00	0.00	0.00	0.00	0.00	0.00	0.00

Al	2.02	2.04	2.05	2.04	2.03	2.04	2.04	1.99	2.03	2.03	2.03	2.03	2.06	2.04	2.02	2.03	2.03	2.04	2.02	2.02
Fe^{3+}	0.00	0.00	0.01	0.00	0.00	0.00	0.00	0.00	0.00	0.00	0.00	0.00	0.00	0.00	0.00	0.00	0.00	0.00	0.00	0.00
Fe^{2+}	1.51	1.48	1.53	1.54	1.58	1.53	1.52	1.54	1.50	1.54	1.52	1.50	1.53	1.51	1.49	1.52	1.52	1.58	1.57	1.58
Mn	0.04	0.04	0.04	0.05	0.05	0.05	0.04	0.04	0.05	0.03	0.03	0.04	0.04	0.04	0.03	0.04	0.04	0.04	0.05	0.05
Mg	0.84	0.81	0.80	0.71	0.76	0.78	0.79	0.71	0.75	0.79	0.79	0.82	0.78	0.75	0.81	0.77	0.78	0.78	0.79	0.78
Ca	0.57	0.60	0.59	0.60	0.56	0.58	0.57	0.65	0.61	0.60	0.59	0.59	0.60	0.64	0.60	0.63	0.61	0.56	0.56	0.58
Na	0.00	0.00	0.00	0.00	0.00	0.00	0.00	0.00	0.00	0.00	0.00	0.00	0.00	0.00	0.00	0.00	0.00	0.00	0.00	0.00
K	0.00	0.00	0.00	0.00	0.00	0.00	0.00	0.00	0.00	0.00	0.00	0.00	0.00	0.00	0.00	0.00	0.00	0.00	0.00	0.00
Cr	0.00	0.00	0.00	0.00	0.00	0.00	0.00	0.00	0.00	0.00	0.00	0.00	0.00	0.00	0.00	0.00	0.00	0.00	0.00	0.00
Sum	7.99	7.98	8.00	7.97	7.99	7.98	7.97	7.97	7.96	7.99	7.98	7.99	7.99	7.98	7.97	7.99	7.98	7.99	7.99	8.00
**Mg#**	0.36	0.36	0.34	0.32	0.33	0.34	0.35	0.32	0.34	0.34	0.34	0.35	0.34	0.34	0.35	0.34	0.34	0.33	0.34	0.33
**Alm**	0.51	0.51	0.52	0.53	0.53	0.52	0.52	0.52	0.52	0.52	0.52	0.51	0.52	0.51	0.51	0.51	0.52	0.53	0.53	0.53
**Gross**	0.19	0.20	0.20	0.21	0.19	0.20	0.19	0.22	0.21	0.20	0.20	0.20	0.20	0.22	0.20	0.21	0.21	0.19	0.19	0.19
**Pyp**	0.28	0.28	0.27	0.25	0.26	0.27	0.27	0.24	0.26	0.27	0.27	0.28	0.27	0.26	0.28	0.26	0.26	0.26	0.27	0.26
**Spess**	0.01	0.01	0.01	0.02	0.02	0.02	0.01	0.01	0.02	0.01	0.01	0.01	0.01	0.01	0.01	0.01	0.01	0.01	0.02	0.02
T_{GC} (℃)	716	733	758	711	668	717	792	839	837	885	856	871	834	890	849	849	848	720	763	752
P_{GCPQ} (kbar)	12.24	12.67	12.98	12.39	11.90	12.43	20.7	24.5	22.3	21.2	20.8	20.1	21.5	22.4	23.4	19.8	21.4	11.88	12.55	11.67
T_{GCK} (℃)	717	749	731	707	642	708	796	836	837	887	861	876	837	893	852	862	853	706	705	740

	Garnet Rim (Retrograde phase-**M3**)		
	Grt-R-4	Grt-R-5	Grt-R-6
SiO_2	38.69	38.61	38.25
TiO_2	0.01	0.04	0.04
Al_2O_3	22.41	22.22	21.81
FeO	24.32	24.32	24.35

MnO	0.71	0.72	0.72
MgO	6.56	6.71	6.99
CaO	7.05	6.87	7.18
Na_2O	0.00	0.00	0.00
K_2O	0.00	0.00	0.00
Cr_2O_3	0.00	0.02	0.03
Total	99.74	99.51	99.37
O	12.00	12.00	12.00
Si	2.99	2.99	2.98
Ti	0.00	0.00	0.00
Al	2.04	2.03	2.00
Fe^{3+}	0.00	0.00	0.00
Fe^{2+}	1.57	1.58	1.58
Mn	0.05	0.05	0.05
Mg	0.76	0.77	0.81
Ca	0.58	0.57	0.60
Na	0.00	0.00	0.00
K	0.00	0.00	0.00
Cr	0.00	0.00	0.00
Sum	7.99	7.99	8.02
**Mg#**	0.33	0.33	0.34
**Alm**	0.53	0.53	0.52
**Gross**	0.20	0.19	0.20
**Pyp**	0.26	0.26	0.27
**Spess**	0.02	0.02	0.02
T_{GC} (℃)	723	739	742
P_{GCPQ} (kbar)	12.22	12.08	12.49
T_{GCK}	698	685	691

(℃)

	Clinopyroxene line1 (rim–core-rim)																			
	Cpx01-1	Cpx01-2	Cpx01-3	Cpx01-4	Cpx01-5	Cpx01-6	Cpx01-7	Cpx01-8	Cpx01-9	Cpx01-10	Cpx01-11	Cpx01-12	Cpx01-13	Cpx01-14	Cpx01-15	Cpx01-16	Cpx01-17	Cpx01-18	Cpx01-19	Cpx01-20
SiO_2	53.69	53.14	52.73	52.86	52.23	52.58	51.85	52.48	52.30	52.35	52.33	52.42	52.18	52.38	52.92	52.72	52.58	53.04	52.66	52.89
TiO_2	0.16	0.15	0.20	0.24	0.22	0.25	0.28	0.27	0.27	0.24	0.25	0.30	0.26	0.26	0.25	0.26	0.20	0.14	0.25	0.18
Al_2O_3	2.10	2.59	2.83	3.02	3.04	2.99	3.20	3.22	3.28	3.28	3.35	3.31	3.31	3.27	3.24	3.20	3.06	2.93	2.81	2.77
FeO	7.27	8.12	8.73	8.49	8.72	9.39	9.66	8.86	8.74	8.80	8.63	8.68	9.01	8.96	9.09	8.90	9.14	8.67	8.45	8.03
MnO	0.06	0.14	0.13	0.07	0.07	0.02	0.10	0.04	0.08	0.05	0.07	0.10	0.08	0.07	0.09	0.09	0.11	0.13	0.07	0.01
MgO	13.21	12.42	12.16	12.07	12.04	12.42	12.16	11.68	11.90	11.77	11.78	11.82	11.98	11.95	12.00	12.00	12.10	12.23	12.50	12.55
CaO	21.75	21.14	20.73	21.23	20.65	20.08	20.33	21.18	20.95	21.18	21.11	21.35	21.10	20.82	21.08	21.07	21.17	21.46	21.57	22.06
Na_2O	0.79	0.95	0.85	0.94	1.04	0.88	1.08	1.00	1.18	1.10	0.97	0.99	1.01	0.97	1.01	0.87	0.86	0.81	0.84	0.70
K_2O	0.01	0.00	0.01	0.00	0.00	0.00	0.00	0.00	0.00	0.01	0.01	0.00	0.00	0.01	0.00	0.00	0.00	0.00	0.00	0.00
Cr_2O_3	0.00	0.02	0.02	0.06	0.06	0.03	0.11	0.07	0.00	0.04	0.01	0.04	0.08	0.03	0.01	0.03	0.00	0.02	0.03	0.03
Total	99.03	98.67	98.38	98.97	98.06	98.65	98.78	98.80	98.71	98.80	98.49	99.01	99.00	98.72	99.68	99.13	99.22	99.42	99.17	99.21
O	6.00	6.00	6.00	6.00	6.00	6.00	6.00	6.00	6.00	6.00	6.00	6.00	6.00	6.00	6.00	6.00	6.00	6.00	6.00	6.00
Si	2.00	1.99	1.99	1.98	1.98	1.98	1.96	1.97	1.97	1.97	1.97	1.97	1.96	1.97	1.97	1.97	1.97	1.98	1.97	1.97
Ti	0.00	0.00	0.01	0.01	0.01	0.01	0.01	0.01	0.01	0.01	0.01	0.01	0.01	0.01	0.01	0.01	0.01	0.00	0.01	0.00
Al	0.09	0.11	0.13	0.13	0.14	0.13	0.14	0.14	0.15	0.15	0.15	0.15	0.15	0.14	0.14	0.14	0.13	0.13	0.12	0.12
Fe^{3+}	0.00	0.00	0.00	0.00	0.00	0.00	0.02	0.00	0.01	0.00	0.00	0.00	0.00	0.00	0.00	0.00	0.00	0.00	0.00	0.00
Fe^{2+}	0.23	0.25	0.27	0.27	0.28	0.30	0.28	0.28	0.26	0.28	0.27	0.27	0.28	0.28	0.28	0.28	0.29	0.27	0.26	0.25
Mn	0.00	0.00	0.00	0.00	0.00	0.00	0.00	0.00	0.00	0.00	0.00	0.00	0.00	0.00	0.00	0.00	0.00	0.00	0.00	0.00
Mg	0.73	0.69	0.68	0.67	0.68	0.70	0.68	0.65	0.67	0.66	0.66	0.66	0.67	0.67	0.67	0.67	0.68	0.68	0.70	0.70
Ca	0.87	0.85	0.84	0.85	0.84	0.81	0.82	0.85	0.84	0.85	0.85	0.86	0.85	0.84	0.84	0.84	0.85	0.86	0.86	0.88
Na	0.06	0.07	0.06	0.07	0.08	0.06	0.08	0.07	0.09	0.08	0.07	0.07	0.07	0.07	0.07	0.06	0.06	0.06	0.06	0.05
K	0.00	0.00	0.00	0.00	0.01	0.01	0.01	0.01	0.01	0.01	0.01	0.01	0.01	0.02	0.02	0.02	0.02	0.02	0.02	0.02
Cr	0.00	0.00	0.00	0.00	0.00	0.00	0.00	0.00	0.00	0.00	0.00	0.00	0.00	0.00	0.00	0.00	0.00	0.00	0.00	0.00
Sum	3.98	3.98	3.98	3.98	3.99	3.98	4.00	3.98	4.00	3.99	3.98	3.99	3.99	3.99	3.99	3.98	3.99	3.98	3.99	3.98
**Mg#**	0.76	0.73	0.71	0.72	0.71	0.70	0.71	0.70	0.72	0.70	0.71	0.71	0.70	0.70	0.70	0.71	0.70	0.72	0.73	0.74

**En**	0.40	0.39	0.38	0.38	0.38	0.39	0.38	0.37	0.37	0.37	0.37	0.37	0.37	0.37	0.37	0.37	0.37	0.38	0.38	0.38
**Fs**	0.12	0.14	0.15	0.15	0.15	0.16	0.17	0.16	0.15	0.15	0.15	0.15	0.16	0.16	0.16	0.16	0.16	0.15	0.14	0.14
**Wo**	0.47	0.47	0.47	0.48	0.47	0.45	0.45	0.48	0.47	0.48	0.48	0.48	0.47	0.47	0.47	0.47	0.47	0.47	0.47	0.48

			Clinopyroxene in garnet (Prograde phase-**M1**)						Clinopyroxene Core (Peak metamorphic phase-**M2**)											
	Cpx01-21	Cpx01-22	Cpx in Grt-1	Cpx in Grt-2	Cpx in Grt-3	Cpx in Grt-4	Cpx in Grt-5	Cpx in Grt-6	Cpx-C-1	Cpx-C-2	Cpx-C-3	Cpx-C-4	Cpx-C-5	Cpx-C-6	Cpx-C-7	Cpx-C-8	Cpx-C-9	Cpx-C-10	Cpx-C-11	Cpx-R-1
SiO_2	52.71	52.15	52.26	52.32	52.07	52.54	53.06	52.45	52.36	51.97	52.28	51.99	51.80	52.00	51.98	51.50	52.55	52.05	52.05	52.74
TiO_2	0.24	0.32	0.32	0.26	0.25	0.26	0.33	0.28	0.26	0.25	0.28	0.35	0.37	0.33	0.34	0.36	0.17	0.34	0.30	0.32
Al_2O_3	2.78	2.92	2.79	2.82	3.60	3.52	2.75	3.10	3.00	3.27	3.36	3.70	3.67	3.60	3.40	3.69	3.03	3.44	3.39	2.76
FeO	7.74	8.52	7.66	8.16	8.06	8.14	7.34	7.87	8.97	9.64	9.07	10.26	9.58	9.54	9.04	10.01	9.15	10.03	9.53	8.37
MnO	0.03	0.02	0.03	0.05	0.01	0.02	0.05	0.03	0.06	0.05	0.08	0.06	0.03	0.07	0.03	0.03	0.13	0.08	0.06	0.02
MgO	12.71	12.77	12.86	12.85	12.59	12.40	13.43	12.83	12.52	12.12	11.74	11.55	11.59	11.63	11.57	11.53	12.20	12.26	11.87	12.55
CaO	22.01	21.94	21.50	21.37	20.53	21.35	21.76	21.30	20.92	19.75	20.93	20.17	20.37	20.74	20.83	20.08	21.87	20.73	20.68	21.39
Na_2O	0.71	0.70	0.90	0.90	0.98	1.06	0.78	0.92	0.79	1.10	1.09	1.10	1.01	1.16	1.10	1.03	0.59	0.81	0.98	0.96
K_2O	0.00	0.01	0.01	0.01	0.00	0.00	0.00	0.00	0.02	0.00	0.01	0.01	0.00	0.00	0.00	0.00	0.00	0.01	0.00	0.00
Cr_2O_3	0.07	0.01	0.01	0.00	0.03	0.00	0.01	0.01	0.00	0.04	0.02	0.02	0.01	0.02	0.03	0.03	0.00	0.06	0.02	0.00
Total	99.00	99.34	98.33	98.72	98.10	99.28	99.50	98.79	98.90	98.18	98.85	99.22	98.42	99.07	98.33	98.26	99.69	99.79	98.88	99.11
O	6.00	6.00	6.00	6.00	6.00	6.00	6.00	6.00	6.00	6.00	6.00	6.00	6.00	6.00	6.00	6.00	6.00	6.00	6.00	6.00
Si	1.97	1.95	1.97	1.96	1.96	1.96	1.97	1.96	1.97	1.97	1.97	1.95	1.96	1.96	1.96	1.95	1.96	1.95	1.96	1.97
Ti	0.01	0.01	0.01	0.01	0.01	0.01	0.01	0.01	0.01	0.01	0.01	0.01	0.01	0.01	0.01	0.01	0.00	0.01	0.01	0.01
Al	0.12	0.13	0.12	0.12	0.16	0.15	0.12	0.14	0.13	0.15	0.15	0.16	0.16	0.16	0.15	0.16	0.13	0.15	0.15	0.12
Fe^{3+}	0.00	0.02	0.01	0.01	0.00	0.00	0.00	0.00	0.00	0.00	0.00	0.01	0.00	0.01	0.00	0.00	0.00	0.01	0.00	0.00
Fe^{2+}	0.24	0.25	0.23	0.25	0.25	0.25	0.23	0.25	0.28	0.31	0.29	0.31	0.30	0.29	0.29	0.32	0.29	0.30	0.30	0.26
Mn	0.00	0.00	0.00	0.00	0.00	0.00	0.00	0.00	0.00	0.00	0.00	0.00	0.00	0.00	0.00	0.00	0.00	0.00	0.00	0.00
Mg	0.71	0.71	0.72	0.72	0.71	0.69	0.74	0.72	0.70	0.68	0.66	0.65	0.65	0.65	0.65	0.65	0.68	0.68	0.67	0.70
Ca	0.88	0.88	0.87	0.86	0.83	0.85	0.86	0.85	0.84	0.80	0.84	0.81	0.82	0.84	0.84	0.82	0.88	0.83	0.83	0.86
Na	0.05	0.05	0.07	0.07	0.07	0.08	0.06	0.07	0.06	0.08	0.08	0.08	0.07	0.08	0.08	0.08	0.04	0.06	0.07	0.07
K	0.02	0.00	0.00	0.00	0.00	0.00	0.00	0.00	0.00	0.00	0.00	0.00	0.00	0.00	0.00	0.00	0.00	0.00	0.00	0.00
Cr	0.00	0.00	0.00	0.00	0.00	0.00	0.00	0.00	0.00	0.00	0.00	0.00	0.00	0.00	0.00	0.00	0.00	0.00	0.00	0.00

Sum	3.99	4.00	4.00	4.00	3.99	3.99	3.99	3.99	3.99	3.99	3.99	3.99	3.99	4.00	3.99	3.99	3.99	4.00	3.99	3.99
**Mg#**	0.75	0.74	0.75	0.74	0.74	0.73	0.76	0.74	0.71	0.69	0.70	0.67	0.68	0.68	0.69	0.67	0.70	0.68	0.69	0.73
**En**	0.39	0.38	0.39	0.39	0.40	0.38	0.40	0.39	0.38	0.38	0.37	0.36	0.37	0.36	0.37	0.37	0.37	0.37	0.37	0.38
**Fs**	0.13	0.14	0.13	0.14	0.14	0.14	0.12	0.14	0.15	0.17	0.16	0.18	0.17	0.17	0.16	0.18	0.16	0.17	0.17	0.14
**Wo**	0.48	0.47	0.47	0.47	0.46	0.47	0.47	0.47	0.46	0.45	0.47	0.46	0.46	0.47	0.47	0.46	0.48	0.45	0.46	0.47
T_{GC} (℃)			716	733	758	711	668	717	792	839	837	885	856	871	834	890	849	849	848	720
P_{GCPQ} (kbar)			12.24	12.67	12.98	12.39	11.90	12.43	20.7	24.5	22.3	21.2	20.8	20.1	21.5	22.4	23.4	19.8	21.4	11.88
T_{GCK} (℃)			717	749	731	707	642	708	796	836	837	887	861	876	837	893	852	862	853	706

	Clinopyroxene Rim (Retrograde phase-**M3**)				
	Cpx-R-2	Cpx-R-3	Cpx-R-4	Cpx-R-5	Cpx-R-6
SiO_2	52.70	52.05	52.33	52.45	52.46
TiO_2	0.30	0.31	0.28	0.30	0.31
Al_2O_3	2.94	3.64	2.78	3.03	3.22
FeO	8.79	8.16	8.74	8.51	8.38
MnO	0.03	0.08	0.06	0.05	0.06
MgO	12.48	11.98	12.53	12.39	12.33
CaO	21.04	21.89	21.04	21.34	22.14
Na_2O	0.92	0.81	0.96	0.91	0.82
K_2O	0.00	0.00	0.00	0.00	0.00
Cr_2O_3	0.00	0.00	0.01	0.00	0.01
Total	99.19	98.92	98.73	98.99	99.72
O	6.00	6.00	6.00	6.00	6.00
Si	1.97	1.95	1.97	1.97	1.95
Ti	0.01	0.01	0.01	0.01	0.01
Al	0.13	0.16	0.12	0.13	0.14

Fe^{3+}	0.00	0.01	0.00	0.01	0.00
Fe^{2+}	0.27	0.26	0.28	0.27	0.26
Mn	0.00	0.00	0.00	0.00	0.00
Mg	0.70	0.67	0.70	0.69	0.68
Ca	0.84	0.88	0.85	0.86	0.88
Na	0.07	0.06	0.07	0.07	0.06
K	0.00	0.00	0.00	0.00	0.00
Cr	0.00	0.00	0.00	0.00	0.00
Sum	3.99	3.99	4.00	3.99	4.00
**Mg#**	0.72	0.72	0.72	0.72	0.72
**En**	0.38	0.37	0.38	0.38	0.37
**Fs**	0.15	0.14	0.15	0.15	0.14
**Wo**	0.46	0.49	0.46	0.47	0.48
T_{GC} (℃)	763	752	723	739	742
P_{GCPQ} (kbar)	12.55	11.67	12.22	12.08	12.49
T_{GCK} (℃)	705	740	698	685	691

	Plagioclase in garnet (Prograde phase-**M1**)						Small Plagioclase around garnet (Retrograde phase-**M3**)					
	Pl in Grt-1	Pl in Grt-2	Pl in Grt-3	Pl in Grt-4	Pl in Grt-5	Pl in Grt-6	Pl out Grt-1	Pl out Grt-2	Pl out Grt-3	Pl out Grt-4	Pl out Grt-5	Pl out Grt-6
SiO_2	58.86	58.80	58.72	59.16	59.77	59.06	58.24	58.39	57.53	58.43	58.14	58.41
TiO_2	0.00	0.00	0.00	0.02	0.05	0.01	0.02	0.00	0.01	0.01	0.01	0.03
Al_2O_3	25.90	25.83	25.78	25.76	25.28	25.71	27.10	26.56	27.45	25.97	26.77	26.95
FeO	0.18	0.12	0.14	0.16	0.33	0.19	0.32	0.30	0.28	0.45	0.34	0.33
MnO	0.00	0.03	0.00	0.00	0.00	0.01	0.02	0.02	0.04	0.00	0.02	0.06
MgO	0.00	0.01	0.00	0.00	0.00	0.00	0.02	0.00	0.00	0.01	0.01	0.00

CaO	7.70	7.75	7.52	7.46	6.77	7.44	7.71	7.59	8.37	7.64	7.83	7.53
Na_2O	7.17	7.20	7.18	7.22	7.49	7.25	7.17	7.27	6.72	7.21	7.09	7.28
K_2O	0.04	0.02	0.02	0.03	0.02	0.03	0.04	0.05	0.04	0.04	0.04	0.03
Total	99.86	99.75	99.36	99.81	99.70	99.70	100.63	100.17	100.44	99.75	100.25	100.61
O	8.00	8.00	8.00	8.00	8.00	8.00	8.00	8.00	8.00	8.00	8.00	8.00
Si	2.63	2.63	2.64	2.64	2.67	2.64	2.59	2.61	2.56	2.62	2.59	2.60
Al	1.36	1.36	1.36	1.36	1.33	1.36	1.42	1.40	1.44	1.37	1.41	1.41
Ti	0.00	0.00	0.00	0.00	0.00	0.00	0.00	0.00	0.00	0.00	0.00	0.00
Fe^{3+}	0.00	0.00	0.00	0.00	0.00	0.00	0.00	0.00	0.00	0.00	0.00	0.00
Fe^{2+}	0.01	0.00	0.01	0.01	0.01	0.01	0.01	0.01	0.01	0.02	0.01	0.01
Mn	0.00	0.00	0.00	0.00	0.00	0.00	0.00	0.00	0.00	0.00	0.00	0.00
Mg	0.00	0.00	0.00	0.00	0.00	0.00	0.00	0.00	0.00	0.00	0.00	0.00
Ca	0.37	0.37	0.36	0.36	0.32	0.36	0.37	0.36	0.40	0.37	0.37	0.36
Na	0.62	0.62	0.62	0.63	0.65	0.63	0.62	0.63	0.58	0.63	0.61	0.63
K	0.00	0.00	0.00	0.00	0.00	0.00	0.00	0.00	0.00	0.00	0.00	0.00
Sum	5.00	5.00	4.99	4.99	4.98	4.99	5.01	5.01	5.00	5.00	5.00	5.01
**XAn**	0.37	0.37	0.37	0.36	0.33	0.36	0.37	0.36	0.41	0.37	0.38	0.36
**XAb**	0.63	0.63	0.63	0.64	0.67	0.64	0.63	0.63	0.59	0.63	0.62	0.64
**XOr**	0.00	0.00	0.00	0.00	0.00	0.00	0.00	0.00	0.00	0.00	0.00	0.00
T_{GC} (℃)	716	733	758	711	668	717	720	763	752	723	739	742
P_{GCPQ} (kbar)	12.24	12.67	12.98	12.39	11.90	12.43	11.88	12.55	11.67	12.22	12.08	12.49
T_{GCK} (℃)	717	749	731	707	642	708	706	705	740	698	685	691

Note: Major element values are in wt.%. Mg# is equal to molar Mg / (Mg + Fe^{2+}). Alm in garnet is equal to molar Fe^{2+} / (Fe^{2+} + Ca + Mg + Mn); Gross is equal to molar Ca / (Fe^{2+} + Ca + Mg + Mn); Pyp is equal to molar Mg / (Fe^{2+} + Ca + Mg + Mn); Spess is equal to molar Mn / (Fe^{2+} + Ca + Mg + Mn). En in clinopyroxene is equal to molar Mg / (Mg + Ca + Fe^{2+}); Fs is equal to molar Fe^{2+} / (Mg + Ca + Fe^{2+}); Wo is equal to molar 100*Ca / (Mg + Ca + Fe^{2+}). Ab in plagioclase is equal to molar Na / (Na + Ca + K); An is equal to molar Ca / (Na + Ca + K); Or is equal to molar K / (Na + Ca + K). The mineral pairs and their calculated P-T conditions using various geothermobarometers for each metamorphic stage are listed.

Table S5. Representative trace elements of garnet and clinopyroxene in the garnet clinopyroxenite from the Shangying rock units of the Zunhua-Shangying ophiolitic mélange, North China Craton.

	Garnet line (rim–core-rim)														Prograde phase (M1)				Peak phase (M2)	
	Grt-01	Grt-02	Grt-03	Grt-04	Grt-05	Grt-06	Grt-07	Grt-08	Grt-09	Grt-10	Grt-11	Grt-12	Grt-13	Grt-14	Cpx-1	Grt-1	Cpx-2	Grt-2	Cpx-3	Grt-3
Li	1.22	1.72	1.55	0.87	1.28	1.01	1.26	1.14	0.95	0.99	1.14	1.22	1.06	1.37	22.28	1.54	25.57	2.55	20.22	1.50
Be	0.00	0.00	0.00	0.00	0.00	0.00	0.00	0.09	0.00	0.00	0.00	0.00	0.00	0.00	0.29	0.00	0.19	0.00	0.38	0.00
B	2.57	1.43	2.86	2.53	1.26	1.78	0.00	2.07	0.20	2.81	0.39	3.51	0.00	0.33	1.81	1.85	1.38	0.00	1.09	4.24
Sc	41.00	42.97	46.23	46.94	49.56	48.12	50.37	51.20	49.57	48.33	47.45	46.45	43.64	40.56	39.84	45.50	43.63	47.86	44.85	46.70
V	156.32	159.98	162.70	166.53	174.12	173.92	171.35	173.75	168.46	173.28	171.13	167.99	157.91	152.91	399.89	217.66	416.46	212.46	463.69	209.05
Cr	293.41	286.65	276.27	265.77	249.68	197.41	170.89	175.92	185.85	214.99	184.97	165.76	263.28	274.75	152.92	423.88	203.73	363.19	197.77	392.13
Co	48.84	49.61	50.48	50.11	51.82	51.14	50.16	49.20	49.99	50.41	49.23	49.54	50.54	47.32	48.80	51.29	46.55	58.19	47.52	52.88
Ni	3.24	3.13	4.11	3.39	2.84	3.89	2.13	4.73	2.78	3.41	3.73	4.22	2.75	2.18	136.61	3.88	121.31	24.14	123.71	4.39
Cu	0.19	0.09	0.29	0.14	0.06	0.39	0.22	0.92	0.04	0.06	0.04	0.00	0.02	0.00	0.19	0.00	0.00	0.00	0.11	0.08
Zn	57.34	59.02	66.52	63.77	58.23	70.39	61.06	66.76	65.61	61.37	70.61	63.50	59.39	51.43	126.93	71.00	124.30	84.80	130.97	73.52
Ga	7.03	8.05	7.06	6.82	6.62	6.84	6.54	7.43	6.71	6.72	6.60	6.24	6.56	6.50	12.92	8.50	11.61	8.30	13.11	7.21
Rb	0.07	0.00	0.04	0.09	0.00	0.04	0.12	0.05	0.00	0.05	0.00	0.02	0.05	0.00	0.00	0.08	0.29	0.02	0.00	0.00
Sr	0.12	0.06	0.14	0.03	0.01	0.01	0.04	0.14	0.05	0.06	0.05	0.12	0.04	0.04	31.38	0.07	28.18	0.97	28.27	0.01
Zr	2.67	3.46	3.84	3.12	3.42	5.06	4.47	3.42	3.91	4.29	5.43	7.28	4.83	3.81	38.46	11.26	36.68	6.80	42.45	6.73
Nb	0.00	0.00	0.00	0.00	0.00	0.00	0.01	0.01	0.00	0.00	0.02	0.01	0.00	0.00	0.01	0.15	0.63	0.05	0.01	0.00
Mo	0.00	0.06	0.00	0.00	0.00	0.06	0.00	0.00	0.00	0.00	0.00	0.00	0.00	0.00	0.03	0.00	0.06	0.00	0.03	0.00
Ag	0.03	0.00	0.00	0.00	0.00	0.00	0.00	0.02	0.00	0.00	0.00	0.04	0.02	0.05	0.02	0.00	0.01	0.01	0.00	0.01
Cd	0.25	0.52	0.00	0.17	0.25	0.00	0.09	0.26	0.00	0.43	0.44	0.00	0.18	0.45	0.19	0.20	0.42	0.06	0.12	0.06
Sn	0.67	0.50	0.96	1.09	0.33	0.16	0.92	0.93	1.01	0.38	0.55	0.87	0.47	0.59	1.32	0.85	1.43	0.38	1.50	0.56

Sb	0.06	0.00	0.00	0.04	0.04	0.08	0.00	0.04	0.07	0.00	0.00	0.04	0.00	0.03	0.00	0.02	0.00	0.04	0.05	0.01
Cs	0.00	0.00	0.01	0.00	0.00	0.00	0.04	0.02	0.03	0.00	0.00	0.00	0.00	0.00	0.00	0.00	0.02	0.02	0.00	0.03
Ba	0.12	0.00	0.08	0.00	0.00	0.04	0.00	0.20	0.08	0.56	0.04	0.08	0.00	0.04	0.15	0.00	1.20	0.40	0.00	0.18
Hf	0.05	0.01	0.03	0.06	0.06	0.09	0.10	0.01	0.08	0.03	0.11	0.11	0.14	0.04	1.63	0.13	1.74	0.10	1.74	0.15
Ta	0.00	0.00	0.00	0.00	0.00	0.00	0.00	0.00	0.00	0.00	0.00	0.00	0.00	0.00	0.00	0.01	0.04	0.00	0.00	0.00
W	0.01	0.01	0.03	0.00	0.00	0.00	0.00	0.00	0.00	0.00	0.00	0.00	0.00	0.00	0.00	0.00	0.01	0.01	0.00	0.00
Hg	0.00	0.00	0.00	0.00	0.00	0.00	0.00	0.00	0.00	0.00	0.00	0.00	0.00	0.00	0.00	0.00	0.00	0.00	0.00	0.00
Tl	0.00	0.00	0.00	0.01	0.00	0.00	0.01	0.00	0.00	0.02	0.02	0.00	0.00	0.01	0.00	0.01	0.01	0.00	0.00	0.00
Bi	0.01	0.00	0.00	0.00	0.00	0.00	0.00	0.00	0.01	0.00	0.01	0.00	0.00	0.00	0.00	0.00	0.01	0.00	0.00	0.01
Pb	0.00	0.00	0.00	0.00	0.01	0.00	0.00	0.01	0.01	0.01	0.00	0.04	0.00	0.01	0.08	0.03	0.05	0.00	0.03	0.01
Th	0.00	0.00	0.01	0.00	0.00	0.00	0.00	0.00	0.00	0.00	0.00	0.00	0.00	0.00	0.00	0.00	0.01	0.00	0.00	0.00
U	0.00	0.00	0.00	0.00	0.00	0.00	0.00	0.00	0.00	0.00	0.00	0.00	0.00	0.00	0.00	0.00	0.00	0.00	0.00	0.00
La	0.00	0.00	0.09	0.00	0.00	0.00	0.00	0.00	0.00	0.00	0.00	0.01	0.00	0.00	1.55	0.00	1.55	0.01	1.40	0.00
Ce	0.04	0.02	0.34	0.03	0.02	0.03	0.04	0.04	0.02	0.02	0.02	0.06	0.02	0.03	7.99	0.03	7.79	0.04	7.31	0.05
Pr	0.01	0.01	0.06	0.02	1.08	0.03	0.02	0.04	0.02	0.04	0.04	0.03	0.02	0.03	1.68	0.03	1.58	0.03	1.54	0.03
Nd	0.51	0.45	0.50	0.58	0.52	0.76	0.58	0.55	0.68	0.57	0.51	0.52	0.69	0.59	9.96	0.59	9.90	0.57	8.97	0.40
Sm	1.18	1.47	1.35	1.38	1.38	1.49	1.48	1.84	1.31	1.41	1.40	1.17	1.72	1.54	3.30	1.54	3.19	1.24	2.98	1.27
Eu	0.71	0.70	0.60	0.72	0.73	0.82	0.74	0.69	0.86	0.70	0.90	0.70	0.73	0.82	0.85	0.82	0.84	0.74	0.72	0.68
Gd	4.17	4.74	4.78	4.49	4.99	5.15	5.20	5.22	4.86	4.38	4.51	4.61	4.36	4.70	2.52	4.70	2.35	4.87	2.38	4.14
Tb	0.88	0.96	1.07	1.13	1.14	1.12	1.17	1.16	1.10	1.13	1.10	1.17	1.05	1.13	0.27	1.13	0.22	0.99	0.26	1.09
Dy	7.41	7.34	8.24	7.99	8.43	8.63	8.72	8.68	8.47	8.46	8.75	8.68	8.45	7.31	0.86	7.31	0.87	7.90	0.91	8.07
Ho	1.52	1.72	1.95	1.98	1.99	1.94	2.11	2.12	2.22	1.95	2.11	2.02	1.87	1.49	0.09	1.45	0.11	1.55	0.09	1.71
Er	4.40	5.16	5.87	5.92	6.03	6.09	5.93	6.08	5.79	5.90	5.73	6.06	5.38	4.20	0.13	2.98	0.11	4.59	0.06	4.44
Tm	0.59	0.71	0.77	0.92	0.88	0.83	0.85	0.90	0.84	0.94	0.98	0.88	0.84	0.61	0.00	0.41	0.02	0.59	0.01	0.59
Yb	4.88	4.87	5.93	5.64	6.10	5.76	6.20	6.46	6.43	6.05	6.10	6.14	5.89	3.88	0.03	3.18	0.06	3.97	0.03	4.50
Lu	0.71	0.77	0.92	0.90	1.02	0.94	0.88	0.88	0.85	0.99	1.00	1.03	0.85	0.54	0.00	0.49	0.00	0.47	0.00	0.61
Y	40.44	45.98	52.61	52.81	53.85	55.86	57.76	57.59	57.51	55.79	55.78	56.70	50.53	40.44	2.03	37.63	2.39	46.13	1.96	47.91
Total REE	27.00	28.92	32.45	31.70	34.30	33.57	33.91	34.65	33.46	32.55	33.14	33.07	31.86	26.87	29.24	24.67	28.60	27.56	26.65	27.58

**（La/Yb）N**	0.00	0.00	0.00	0.00	0.00	0.00	0.00	0.00	0.00	0.00	0.00	0.00	0.00	0.00	32.75	0.00	17.16	0.00	39.50	0.00
**（La/Sm）N**	0.00	0.00	0.00	0.00	0.00	0.00	0.00	0.00	0.00	0.00	0.00	0.00	0.00	0.00	0.30	0.00	0.31	0.00	0.30	0.00
**（Gd/Yb）N**	0.71	0.81	0.67	0.66	0.68	0.74	0.69	0.67	0.63	0.60	0.61	0.62	0.61	1.00	61.61	1.22	30.05	1.01	77.51	0.76
**(Eu/Eu*)**	0.87	0.74	0.64	0.81	0.75	0.81	0.73	0.64	0.93	0.79	0.99	0.81	0.77	0.86	0.86	0.86	0.89	0.81	0.80	0.83
**(Ce/Ce*)**	0.89	0.50	0.68	0.50	0.30	0.35	0.82	0.30	0.34	0.14	0.15	0.61	0.25	0.29	1.08	0.29	1.10	0.35	1.08	0.54
T_{GC-REE} (℃)															730±20	730±20	706±41	706±41	844±16	844±16
P_{GC-REE} (kbar)															11.8±0.2	11.8±0.2	11.3±0.4	11.3±0.4	22.3±0.2	22.3±0.2

	Peak phase (M2)		Retrograde phase (M3)			
	Cpx-4	Grt-4	Cpx-5	Grt-5	Cpx-6	Grt-6
Li	24.45	1.36	13.76	1.50	21.11	1.22
Be	0.26	0.00	0.21	0.00	0.25	0.00
B	2.16	1.23	2.73	0.74	1.69	2.57
Sc	41.44	47.81	47.09	43.64	38.47	41.00
V	450.67	205.40	565.64	210.80	636.81	156.32
Cr	160.75	395.97	188.47	379.64	225.55	293.41
Co	46.68	53.56	46.82	48.15	44.91	48.84
Ni	144.35	3.68	168.28	3.25	154.64	3.24

Cu	0.01	0.07	0.40	0.37	0.02	0.19
Zn	133.35	75.55	150.05	50.36	136.45	57.34
Ga	13.41	8.26	14.84	8.33	16.94	7.03
Rb	0.00	0.01	0.00	0.00	0.00	0.07
Sr	32.80	0.04	26.21	2.54	18.26	0.12
Zr	35.95	13.34	54.23	4.87	34.59	2.67
Nb	0.01	0.46	0.00	0.00	0.02	0.00
Mo	0.00	0.00	0.02	0.00	0.00	0.00
Ag	0.04	0.03	0.01	0.04	0.02	0.03
Cd	0.05	0.33	0.00	0.13	0.28	0.25
Sn	1.32	0.79	1.46	0.81	1.61	0.67
Sb	0.02	0.02	0.02	0.00	0.05	0.06
Cs	0.01	0.02	0.00	0.02	0.01	0.00
Ba	0.00	0.03	0.10	0.00	0.34	0.12
Hf	1.69	0.24	2.02	0.08	1.88	0.05
Ta	0.00	0.00	0.00	0.00	0.00	0.00
W	0.00	0.01	0.01	0.00	0.00	0.01
Hg	0.00	0.00	0.00	0.00	0.00	0.00
Tl	0.00	0.00	0.00	0.00	0.01	0.00
Bi	0.01	0.00	0.01	0.00	0.00	0.01
Pb	0.07	0.02	0.04	0.03	0.04	0.00
Th	0.00	0.00	0.00	0.00	0.00	0.00
U	0.00	0.00	0.00	0.00	0.00	0.00
La	1.40	0.04	0.91	0.00	1.42	0.00
Ce	7.53	0.10	5.67	0.06	7.68	0.04
Pr	1.46	0.05	1.22	0.03	1.42	0.01
Nd	9.60	0.64	8.55	0.66	10.46	0.51
Sm	2.76	1.49	2.80	1.20	3.00	1.18
Eu	0.83	0.80	0.70	0.68	0.97	0.71

Gd	2.48	5.10	1.95	4.16	2.70	4.17
Tb	0.28	1.08	0.16	0.85	0.20	0.88
Dy	0.87	8.55	0.62	6.59	0.72	7.41
Ho	0.08	1.73	0.06	1.35	0.07	1.52
Er	0.17	4.24	0.04	3.97	0.07	4.40
Tm	0.02	0.59	0.01	0.51	0.01	0.59
Yb	0.09	4.19	0.02	3.74	0.05	4.88
Lu	0.01	0.46	0.00	0.55	0.01	0.71
Y	2.13	49.94	1.27	37.63	1.73	40.44
Total REE	27.55	29.05	22.72	24.33	28.77	27.00
**（La/Yb）N**	11.78	0.01	39.28	0.00	19.91	0.00
**（La/Sm）N**	0.33	0.02	0.21	0.00	0.31	0.00
**（Gd/Yb）N**	24.09	1.01	97.28	0.92	43.66	0.71
**(Eu/Eu*)**	0.95	0.79	0.86	0.83	1.03	0.87
**(Ce/Ce*)**	1.16	0.46	1.11	0.65	1.20	0.89
$T_{GC\text{-}REE}$ (℃)	875±38	875±38	749±23	749±23	719±28	719±28
$P_{GC\text{-}REE}$(kbar)	21.2±0.4	21.2±0.4	12.7±0.3	12.7±0.3	10.7±0.3	10.7±0.3

SI References

1. Q. X. Xia, Y. F. Zheng, 2011. The composition and chemical zoning in garnet from high to ultra-high pressure metamorphic rocks. *Acta Petrol. Sin.* **27**, 433–450 (2011). (in Chinese with English abstract).

2 F. S. Spear, The duration of near-peak metamorphism from diffusion modelling of garnet zoning. *J. Metamorph. Geol.* **32**, 903–914 (2014).

3 N. Morimoto, Nomenclature of Pyroxenes. *Miner. Petrol.* **39**, 55–76 (1988).

4 E. K. Ravna, The garnet–clinopyroxene Fe2+–Mg geothermometer: an updated calibration. *J. Metamorph. Geol.* **18**, 211–219 (2000).

5 E. J. Krogh, The garnet–clinopyroxene Fe–Mg geothermometer—a reinterpretation of existing experimental data. *Contrib. Miner. Petrol.* **99**, 44–48 (1988).

6 J. O. Eckert, R. C. Newton, O .J. Kleppa, The Δ H of reaction and recalibration of garnet-pyroxene-plagioclase-quartz geobarometers in the CMAS system by solution calorimetry. *Am. Mineral.* **76**, 148–160 (1991).

7 C. F. Beyer, D. J. Frost, N. Miyajima, Experimental calibration of a garnet–clinopyroxene geobarometer for mantle eclogites. *Contrib. Miner. Petrol.* **169**, 1–21 (2015).

8 C. G. Sun, Y. Liang, A REE-in-garnet–clinopyroxene thermobarometer for eclogites, granulites and garnet peridotites. *Chem. Geol.* **393-394**, 79–92 (2015).

9 G. Rossi, D. C. Smith, L. Ungaretti, M. C. Domeneghetti, Crystal-chemistry and cation ordering in the system diopside-jadeite: A detailed study by crystal structure refinement. *Contrib. Miner. Petrol.* **83**, 247–258 (1983).

10 A. Raheim, D. H. Greeen, Experimental determination of the temperature and pressure dependence of the Fe-Mg partition coefficient for coexisting garnet and clinopyroxene. *Contrib. Miner. Petrol.* **48**, 179–203 (1974).

11 T. Mori, D. H. Green, Laboratory Duplication of Phase Equilibria Observed in Natural Garnet Lherzolites. *J. Geol.* **86** (1978).

12 D. J. Ellis, D. H. Green, An experimental study of the effect of Ca upon garnet–clinopyroxene Fe–Mg exchange equilibria. *Contrib. Miner. Petrol.* **71**, 13–22 (1979).

13 Y. Chen, K. Ye, C. M. Wu, Reviews on applying common-used geothermobarometers for eclogite. *Acta Petrol. Sin.* **21**,1067–1080 (2005). (in Chinese with English abstract).

14 L. W. McKenna, K. V. Hodges, Accuracy versus precision in locating reaction boundaries; implications

for the garnet-plagioclase-aluminum silicate-quartz geobarometer. *Am. Mineral.* **73**, 1205–1208 (1988).

15 H. Y. C. Wang, *et al.,* Tectonic mélange records the Silurian–Devonian subduction-metamorphic process of the southern Dunhuang terrane, southernmost Central Asian Orogenic Belt. *Geology* **45**, 427–430 (2017).

16 H. Y. C. Wang, *et al.,* Paleozoic subduction of the southern Dunhuang Orogenic Belt, northwest China: metamorphism and geochronology of the Shuixiakou area. *Geochim. Acta* **30**, 63–83 (2018).

17 D. J. Cherniak, A. Dimanov, Diffusion in pyroxene, mica and amphibole. *Rev. Mineral. Geochem.***72**, 641–690 (2010).

18 A. V. Korsakov, M. J. Kohn, M. Perraki, Applications of Raman Spectroscopy in Metamorphic Petrology and Tectonics. *Elements* **16**, 105–110 (2020).

19 F. Nehring, S. F. Foley, P. Hölttä, Trace element partitioning in the granulite facies. *Contrib. Miner. Petrol.* **159**, 493–519 (2010).

20 A. P. Gysi, O. Jagoutz, M. W. Schmidt, K. Targuisti, Petrogenesis of pyroxenites and melt infiltrations in the ultramafic complex of Beni Bousera, Northern Morocco. *J. Petrol.* **52**, 1679–1735 (2011).

21 Y. Liang, C. G. Sun, L. J. Yao, A REE-in-two-pyroxene thermometer for mafic and ultramafic rocks. *Geochim. Cosmochim. Acta* **102**, 246–260 (2013).

22 C. Yang, C. J. Wei, Ultrahigh temperature (UHT) mafic granulites in the East Hebei, North China Craton: Constraints from a comparison between temperatures derived from REE-based thermometers and major element-based thermometers. *Gondwana Res.* **46**, 156–169 (2017).

23 E. J. K. Ravna, J. Paquin, in *European Mineralogical Union Notes in Mineralogy* (ed D.A. Carswell, Compagnoni, R., Rolfo, F.) Ch. 8, 229–259 (2003).

24 T. Andersen, Correction of common lead in U-Pb analyses that do not report 204Pb. *Chem. Geol.* **192** (1-2): 59–79 (2002).

25 Y. S. Wan, *et al.,* U–Th–Pb behavior of zircons under high-grade metamorphic conditions: A case study of zircon dating of meta-diorite near Qixia, eastern Shandong. *Geosci. Front.* **2**, 137–146 (2011).

26 F. Corfu, J. M. Hanchar, P. W. O. Hoskin, P. Kinny, Atlas of zircon textures. *Rev. Mineral. Geochem.* **53**, 469–500 (2003).

27 Y. B. Wu, Y. F. Zheng, Genesis of zircon and its constraints on interpretation of U-Pb age. *Chin. Sci. Bull.* **49**, 1554–1569 (2004).

28 Y. B. Wu, *et al.* Zircon U-Pb ages and Hf isotope compositions of migmatite from the North Dabie

terrane in China: constraints on partial melting. *J. Metamorph. Geol.* **25**, 991–1009 (2007).

29. T. M. Kusky, J. H. Li, Origin and emplacement of Archean ophiolites of the central orogenic belt, North China craton. *J. Earth Sci.* **21**, 744–781 (2010).

30. J. J. Ague, J. O. Eckert, Precipitation of rutile and ilmenite needles in garnet: Implications for extreme metamorphic conditions in the Acadian Orogen, U.S.A. *Am. Mineral.* **97**, 840–855 (2012).

31. S. S. Sun, W. F. McDonough, Chemical and isotopic systematics of oceanic basalts: implications for mantle composition and processes. *Geol. Soc. London Spec. Publ.* **42**, 313–345 (1989).

32. E. A. K. Middlemost, Naming materials in the magma/igneous rock system. *Earth Sci. Rev.* **37**, 215–224 (1994).

33. T. N. Irvine, W. R. A. Baragar, A Guide to the Chemical Classification of the Common Volcanic Rocks. *Can. J. Earth Sci.* **8**, 523–548 (1971).

34. J. A. Pearce, Geochemical fingerprinting of oceanic basalts with applications to ophiolite classification and the search for Archean oceanic crust. *Lithos* **100**, 14–48 (2008).

35. M. Meschede, A method of discriminating between different types of mid–ocean ridge basalts and continental tholeiites with the Nb–Zr–Y diagram. *Chem. Geol.* **56**, 207–218 (1986).

36. H. Downes, Origin and significance of spinel and garnet pyroxenites in the shallow lithospheric mantle: Ultramafic massifs in orogenic belts in Western Europe and NW Africa. *Lithos* **99**, 1-24 (2007).

37. S. Y. Yu *et al.,* Remnants of oceanic lower crust in the subcontinental lithospheric mantle: Trace element and Sr–Nd–O isotope evidence from aluminous garnet pyroxenite xenoliths from Jiaohe, Northeast China. *Earth Planet. Sci. Lett.* **297**, 413–422 (2010).

38. B. Huang *et al.,* Paired metamorphism in the Neoarchean: a record of accretionary to–collisional orogenesis in the North China Craton. *Earth Planet. Sci. Lett.* **543**, 116355 (2020).

39. W. B. Ning *et al.,* From subduction initiation to arc–polarity reversal: Life cycle of an Archean subduction zone from the Zunhua ophiolitic mélange, North China Craton. *Precambr. Res.* **350**, 105868 (2020).

40. S. R. Taylor, S. M. McLennan, The Continental Crust: Its Composition and Evolution, (Blackwell, Oxford, 1985), pp. 85–86.
